# FAVIS: Fast and versatile protocol for non-destructive metabarcoding of bulk insect samples

**DOI:** 10.1371/journal.pone.0286272

**Published:** 2023-07-19

**Authors:** Elzbieta Iwaszkiewicz-Eggebrecht, Piotr Łukasik, Mateusz Buczek, Junchen Deng, Emily A. Hartop, Harald Havnås, Monika Prus-Frankowska, Carina R. Ugarph, Paulina Viteri, Anders F. Andersson, Tomas Roslin, Ayco J. M. Tack, Fredrik Ronquist, Andreia Miraldo

**Affiliations:** 1 Department of Bioinformatics and Genetics, Swedish Museum of Natural History, Stockholm, Sweden; 2 Institute of Environmental Sciences, Faculty of Biology, Jagiellonian University, Kraków, Poland; 3 Doctoral School of Exact and Natural Sciences, Jagiellonian University, Kraków, Poland; 4 Station Linné, Färjestaden, Sweden; 5 Center for Integrative Biodiversity Discovery, Museum für Naturkunde—Leibniz Institute for Evolution and Biodiversity Science, Berlin, Germany; 6 Science for Life Laboratory, Department of Gene Technology, KTH Royal Institute of Technology, Stockholm, Sweden; 7 Department of Ecology, Swedish University of Agricultural Sciences, Uppsala, Sweden; 8 Department of Ecology, Environment and Plant Sciences, Stockholm University, Stockholm, Sweden; University of Helsinki: Helsingin Yliopisto, FINLAND

## Abstract

Insects are diverse and sustain essential ecosystem functions, yet remain understudied. Recent reports about declines in insect abundance and diversity have highlighted a pressing need for comprehensive large-scale monitoring. Metabarcoding (high-throughput bulk sequencing of marker gene amplicons) offers a cost-effective and relatively fast method for characterizing insect community samples. However, the methodology applied varies greatly among studies, thus complicating the design of large-scale and repeatable monitoring schemes. Here we describe a non-destructive metabarcoding protocol that is optimized for high-throughput processing of Malaise trap samples and other bulk insect samples. The protocol details the process from obtaining bulk samples up to submitting libraries for sequencing. It is divided into four sections: 1) Laboratory workspace preparation; 2) Sample processing—decanting ethanol, measuring the wet-weight biomass and the concentration of the preservative ethanol, performing non-destructive lysis and preserving the insect material for future work; 3) DNA extraction and purification; and 4) Library preparation and sequencing. The protocol relies on readily available reagents and materials. For steps that require expensive infrastructure, such as the DNA purification robots, we suggest alternative low-cost solutions. The use of this protocol yields a comprehensive assessment of the number of species present in a given sample, their relative read abundances and the overall insect biomass. To date, we have successfully applied the protocol to more than 7000 Malaise trap samples obtained from Sweden and Madagascar. We demonstrate the data yield from the protocol using a small subset of these samples.

## Introduction

Insects are key players in ecosystems—they are a crucial part of food webs and provide a wealth of ecosystem functions and services. They are therefore indispensable for the maintenance of natural systems as well as for food production [1]. Insects are also highly diverse with estimates ranging from 4 to 7 million species, which makes them one of the most species-rich groups of animals on Earth [2–4]. However, despite insects’ tremendous diversity and ecological importance, our knowledge about them is still fragmentary, and an estimated 75 to 85% of insect species still remain undescribed [5]. Worryingly, recent studies of trends in insect abundance and diversity [6–8] have raised alarm about worldwide insect declines and subsequent threats to the stability of terrestrial ecosystems [9]. Thus, there is a pressing need to speed up efforts in insect diversity discovery and monitoring.

Traditional methods used to study and describe insects involve collecting specimens with a range of different traps, followed by sorting and classification of samples into taxonomic fractions. Whilst the first part of this process—sampling—is relatively straightforward and can be performed by volunteers [10], the latter—taxonomic identification—is complex, demanding specialized knowledge which is in short supply, and can be incredibly time-consuming. For instance, *the Swedish Malaise Trap Project*—an ambitious project aiming to characterize the entire insect fauna of Sweden—collected 1919 bulk insect community samples, containing an estimated 20 million individuals, over three years. Sorting those samples into some 350 taxonomic fractions suitable for processing by specialists took 15 years, despite a considerable investment in manpower [11]. Furthermore, the material identified to species level accounted for only 1% of the total specimen number [12]. In another study, the mapping of insect diversity in a single tropical forest took a decade, and involved the work of 110 taxonomists [13]. Such delays in obtaining results hamper the development of meaningful conservation or protection measures in a timely fashion. For adequate insect diversity discovery, insect community monitoring and real-time study of the spatio-temporal dynamics of insect communities, it is imperative that we develop high-throughput methods for taxonomic processing of insect samples.

DNA-based methods appear particularly well suited to address these high-throughput needs [14]. Reference databases are constantly growing and the cost of sequencing is decreasing, adding to their appeal. Methods such as metagenomics or genome skimming–i.e., filtering of high-copy loci, such as mitochondria, chloroplasts or rRNA, mitochondrial sequences after sequencing–can provide high taxonomic resolution and even promise to provide accurate abundance estimates from bulk samples [15, 16]. However, these techniques still remain prohibitively expensive for most large-scale insect monitoring projects. Metabarcoding—i.e., the amplification of large numbers of barcode sequences from bulk samples—is a cost-effective alternative that has gained popularity in recent years [17–19] and has been successfully applied in arthropod community surveys [14, 20, 21]. Metabarcoding relies on the use of the DNA barcoding technique, developed by Hebert and colleagues [22, 23], in which a short DNA fragment of an individual (i.e., a barcode) can provide us with species-level identification. The standard barcode used in eukaryotic diversity studies is the Folmer region [24] of the mitochondrial cytochrome *c* oxidase subunit 1 (COI) gene, for which vast reference databases exist [25]. In metabarcoding studies, DNA is extracted from bulk, multi-species samples (as derived from e.g. a Malaise trap, or a water or soil sample). Then barcodes are amplified via PCR, sequenced and compared to the reference database for taxonomic identification. The species can be named by matching the barcode to a reference database, providing that the species is represented in the database. Due to high insect diversity and large knowledge gaps, certain taxonomic groups are poorly represented among the references—both because of the lack of voucher material for described species, and because of a high proportion of undescribed species. Both aspects will contribute to lowering the success of species-level identification. Nevertheless, even for those poorly represented groups, it is still possible to group sequences into clusters based on their genetic similarity, obtain taxonomic assignment for these clusters at higher levels (i.e., order, family or genus), and compare their presence among samples–thereby allowing the efficient characterization of the community composition of the original sample collection.

Despite the great potential of metabarcoding, many methodological questions concerning early stages of sample processing remain open. Perhaps most importantly, the operating procedures for large-scale insect monitoring projects remain motley and poorly documented. In recent years, many different protocols have emerged. Some advocate destructive DNA extraction methods like homogenizing specimens into an “insect soup” [18, 26–28]. Others propose non-destructive mild lysis treatments, in which insects soak in a buffer, gradually releasing their DNA, with minimal damage to specimens [19, 29–31]. The mild lysis treatment yields smaller DNA amounts [32] but is less laborious and preserves specimens for future molecular and taxonomic work [33–36]. Furthermore, it was recently shown that mild lysis also decreases the rate of false negatives during metabarcoding, as the capability to detect small specimens is improved [32].

Each laboratory and institution has to design a workflow best fitting their aims and needs. To aid those searching for a versatile and scalable solution for their purposes, here we present a complete metabarcoding protocol, from insect bulk samples to sequencing data, initially designed for a large-scale insect monitoring project—the *Insect Biome Atlas* (www.insectbiomeatlas.org). The project’s field campaign took place in Sweden and Madagascar over 12 months during 2019–2020, and yielded 7398 insect community samples collected with Malaise traps, each sample typically representing one week. All samples were processed using this protocol within 12 months. When adapted and optimized, the wet-lab protocol allows one lab technician to process 180 insect community samples from bulk samples to submission for sequencing in one week, allowing the timely delivery of results.

The use of the protocol and further bioinformatic processing result in a dataset that can be used to produce comprehensive lists of species present in a sample, their relative read abundances, and the overall insect biomass. In defining the protocol, we made efforts to reduce costs and adopt universal reagents and materials that can be easily obtained worldwide. For steps that remain costly or inaccessible, such as DNA purification robots, we suggest alternative low-cost solutions when possible. We opted for a non-destructive lysis protocol with a short incubation time (2h 45 min) in a mild lysis buffer [37] as this allows the efficient processing of a large number of samples per day whilst maximizing the power to recover the original species composition of each sample [32]. In order to introduce a correction factor and allow more accurate estimates of species’ abundances, we added to each sample a pre-defined number of biological spike-ins—size-standardized insect species that do not occur in our sampled area (e.g., in the processing of Swedish Malaise trap collection we selected six tropical species that have never been detected in Sweden or neighboring countries). Furthermore, we minimize the damage to specimens and preserve the insect material for further taxonomic or molecular work by returning them to ethanol immediately after the lysis step. Another important aspect of the protocol is the fact that insects never leave the collection bottle, minimizing the risk of cross-sample contamination during sample processing and DNA extraction. The two-step-PCR strategy for COI amplicon library preparation results in double-uniquely indexed libraries obtained using broad-spectrum BF3-BR2 primers [38] with variable-length inserts (phased), reducing cross-contamination through index hopping and increasing signal complexity within the sequencing lane, thus translating to higher quality of results [39].

## Materials and methods

The protocol described in this article is published on protocols.io https://www.protocols.io/private/C609E2107CD8B7CFF46EFF1461DBE4C3 and is included for printing as S1 File with this article.

**Fig 1:**
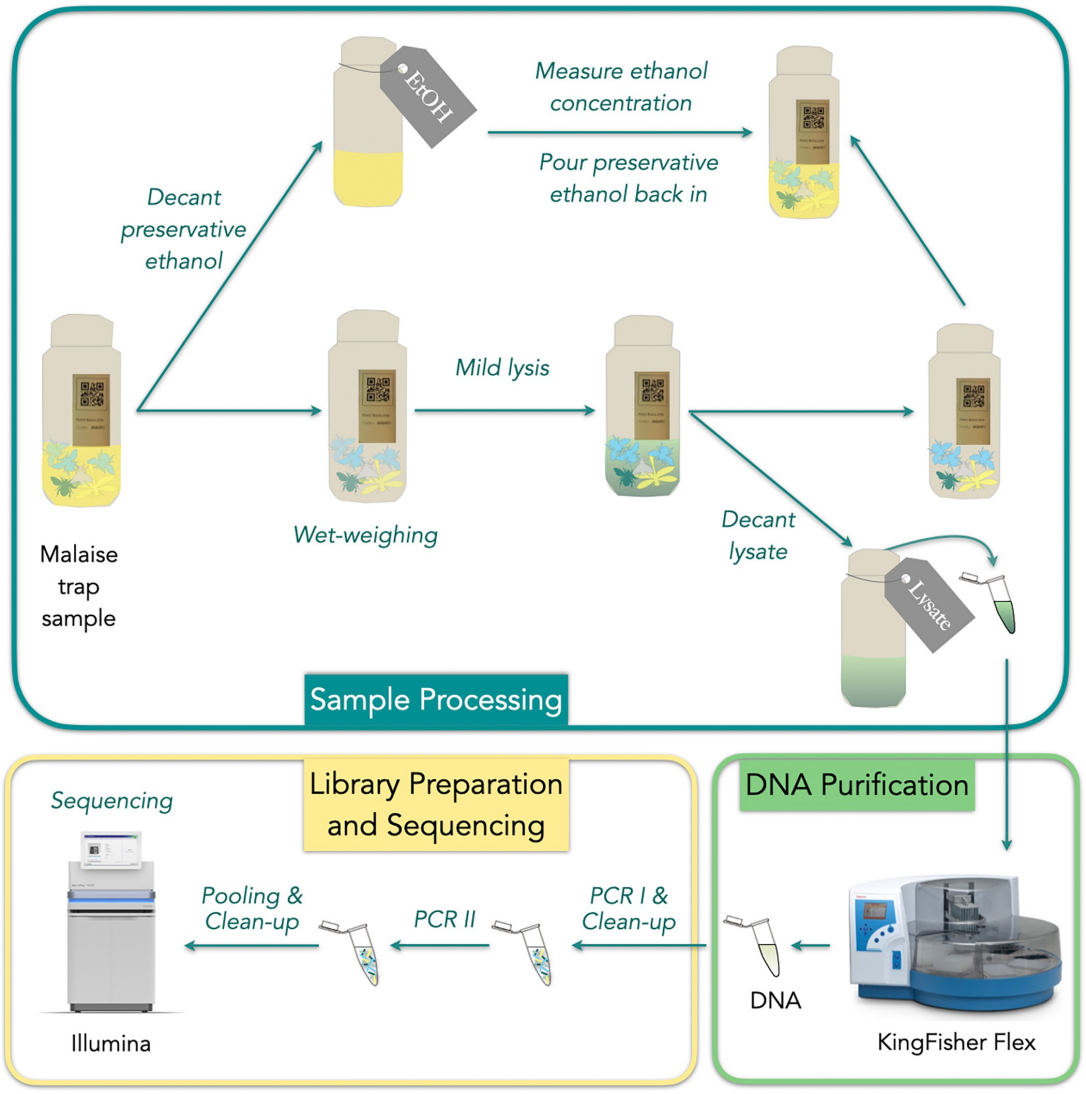
Schematic workflow of FAVIS metabarcoding protocol, comprising three main sections. Sample processing consists of decanting ethanol, measuring ethanol concentration, wet-weighing the sample, adding lysis buffer, incubating the sample, decanting lysates, taking lysate aliquot, and refilling the insect community sample with the previously decanted ethanol. DNA is then extracted and purified from the lysate aliquot with magnetic beads and subsequently used as a template for the amplification of the target COI fragment via PCR (PCR I). After amplification, PCR products are cleaned using magnetic beads and used as template in the second round of PCR (PCR II), where sample-specific tags are added and Illumina adapters completed. The concentration of PCR II products is assessed based on the band brightness on an agarose gel, and all samples are pooled approximately equimolarly to form the sequencing pool. Finally, the pool is purified with magnetic beads and then sequenced on an Illumina NovaSeq platform.

The protocol is divided into four sections. Section 1 (*Preparation*) describes how to prepare workspace and equipment before starting to process samples. The remaining three sections—sections 2 (*Sample Processing*), 3 (*DNA Purification*) and 4 (*Library Preparation and Sequencing*)—cover the main parts of the protocol **(Fig 1)**.

To demonstrate the utility of the protocol we summarize the sequence data obtained by processing fifteen Malaise trap samples representing three different habitats in Southern Sweden: a forest, a wetland, and a grassland. From each of these habitats, we present data from samples collected during five consecutive weeks between April and May 2019. The 15 samples presented here were processed as part of our high-throughput sample processing, which involved processing 180 bulk insect samples per week. After completing all steps of the protocol and sequencing on an Illumina NovaSeq 6000 SPrime flow cell, sequencing data was processed bioinformatically following pipelines that can be accessed via links: https://github.com/biodiversitydata-se/amplicon-multi-cutadapt (read trimming and filtering); https://nf-co.re/ampliseq (ASV reconstruction and taxonomic annotation). In short, we use c*utadapt v*.*3*.*2* [40] for primer trimming and *R* package *DADA2 v*.*4*.*2*.*1* for denoising [41]. Then we use *SINTAX* [42] in order to get the taxonomic assignment for all ASVs using a custom-made reference COI database (https://doi.org/10.17044/scilifelab.20514192.v4). Krona plots were prepared with the *q2-krona* plug-in from the *qiimeII v*.*2022*.*2* library [43, 44]. Visualizations of the results were done with *ggplot2 v*.*3*.*4*.*1* [45] and *ggvenn v*.*0*.*1*.*9* [46] packages in the *R* environment [47]. Non-metric multidimensional scaling (nMDS) was calculated using the metaMDS function from the *vegan v*.*2*.*6–4* package [48]. Code used for data manipulation and plotting of the results as well as interactive Krona plots are available on GitHub under https://github.com/ela-iwaszkiewicz/Lab_protocols.git.

### Ethics statement

Samples used in this study were covered by Sweden´s right of access to private land (Allemansrätten) and did not necessitate a collection permit. More information about utilizing Swedish genetic resources can be found at the Swedish Environmental Protection Agency website: https://www.naturvardsverket.se/en/guidance/species-protection/utilizing-genetic-resources

## Results

**Fig 2:**
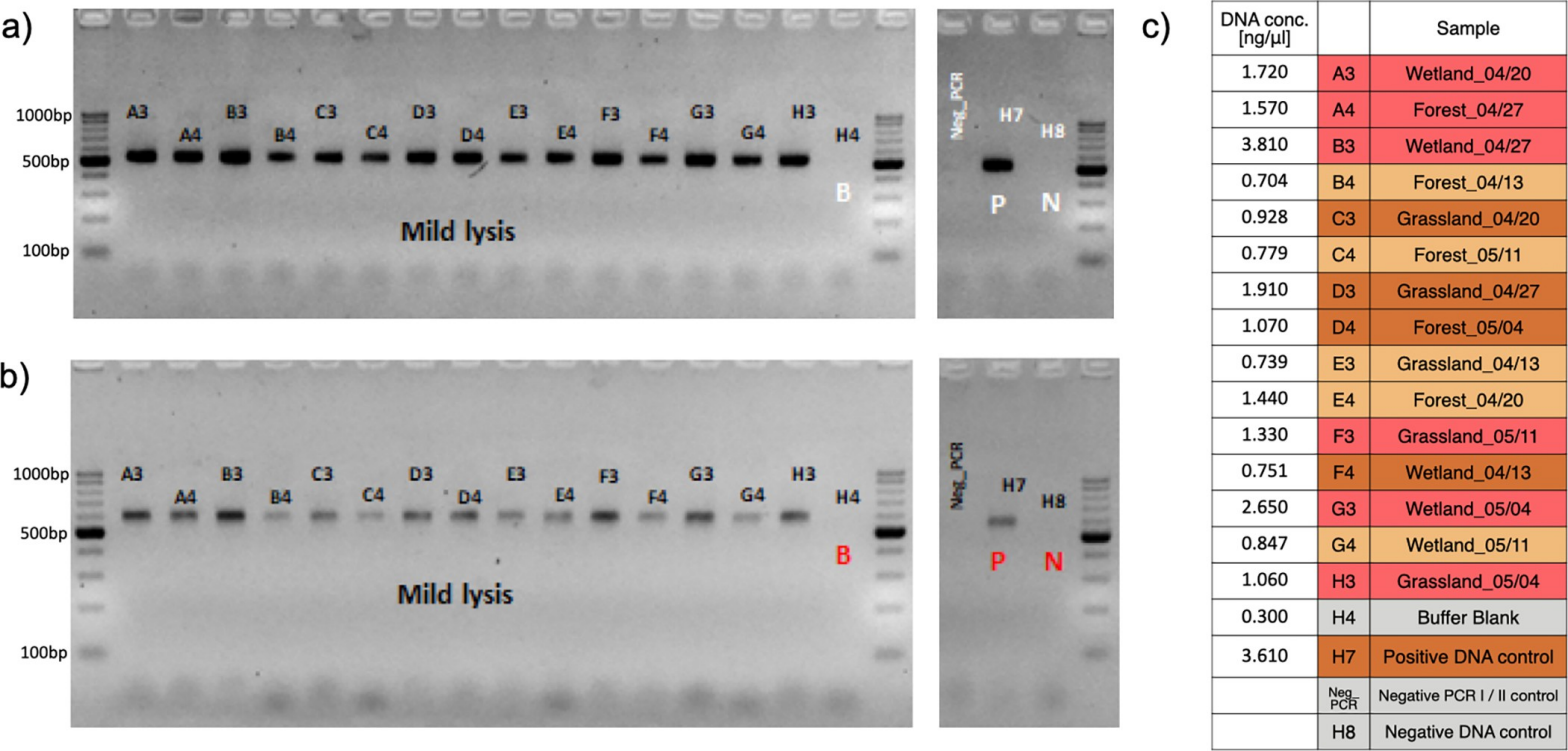
Electrophoresis gel pictures of PCR products. Panel a) shows products after PCR I and b) after PCR II. Panel c) provides sample codes; cell color represent manually assigned PCR II band brightness scores, which determined the amount of product included in the library pool for sequencing (coral = strong = 1μl, brown = medium = 2μl, beige = weak = 4μl, gray = empty = 8μl). The length of the PCR I product was ca. 490bp and of PCR II product, ca. 563bp. Gel was 2.5% agarose and the DNA ladder used was Perfect Ladder 100-1000bp (EurX, Poland). Original, uncropped gel pictures are provided in S1 and S2 Figs.

**Fig 3:**
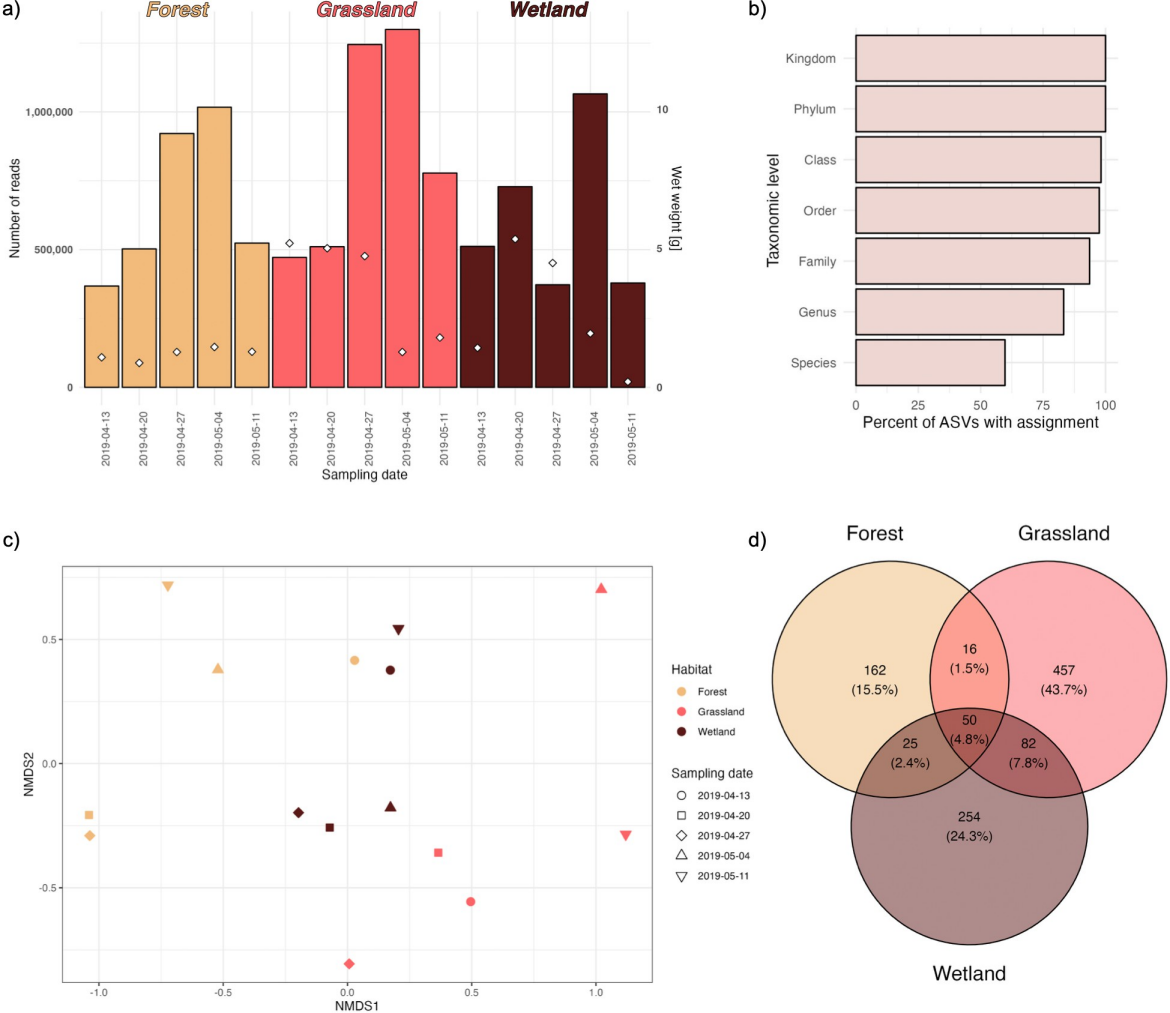
Visual summary of sequencing results. **a)** Wet weight and sequencing depth for 15 samples. The bars show the number of COI reads that passed our quality filters and were used for further analyses. Colors indicate habitat and white diamonds represent sample wet weight (scale shown on the right y-axis). **b)** Percentages of ASVs with taxonomic assignment at a given rank. **c)** Non-metric multidimensional scaling (nMDS) plot based on pairwise Bray-Curtis distances calculated from relative abundances of ASV sequences.Stress factor associated with the ordination is 0.08. **d)** Venn-diagram displaying numbers and percentages of ASVs unique to the different habitats as well as shared between habitats. Note that the data includes seven ASVs representing the biological spike-in species which are shared between all samples. Therefore, the real number of ASVs shared between habitats is lower, i.e. 43 ASVs.

The wet weight of the collected insect material ranged from 0.2 to 5.3 g **(Fig 3A).** Concentration of the DNA purified from lysates ranged from 0.7 to 3.8 ng/μl **(Fig 2C)**. PCR reactions were successful for all samples included in this study as well as for the positive DNA control (qualitative control of the DNA purification step). Negative controls of PCR I, PCR II, DNA extraction and sample processing (buffer blank) did not show detectable contamination in gel electrophoresis **(Fig 2A and 2B)**. In order to even out the amount of each library in the sequencing pool, samples were divided into four categories based on the PCR II product band brightness **(Fig 2C)**.

After filtering out amplicon sequence variants (ASVs) that did not have the correct primer sequences, had stop codon(s) in the expected reading frame (possible nuclear mitochondrial DNA segments, i.e. NUMTs), or were of bacterial origin, we ended up with a total of 10,697,001 read-pairs (2 x 250bp). This amounted to an average of 713,133 verified read-pairs per sample (range: 368,060–1,299,458) **(Fig 3A)**. These reads represented 1,038 ASVs. For 93.6% of these we obtained taxonomic assignments at least at the family level, for 83.2% at the genus level and for 59.7% at the species level **(Fig 3B)**. PCR I and PCR II negative controls did not yield any sequencing results. ASVs detected in the sample processing negative control (buffer blank) were inspected but not excluded from the results. In large-scale processing, we recommend using multiple negative controls—i.e. one buffer blank per each day of sample processing, one DNA negative control per extraction batch and one negative control for each PCR reaction plate—and then excluding from the entire dataset all ASVs that are found in more than 5% of negative controls.

Most of the ASVs were found in only one habitat, with Grassland having the highest barcode richness **(Fig 3D)**. Multivariate ordination methods, such as nMDS, allow visual inspection of the data [49]. Here, the distance between different points—samples—reflect their relative similarities and differences in terms of the ASV read counts. The further the samples are situated from one another the more dissimilar they are. In our dataset, the multidimensional ordination of the samples based on beta-diversity (Bray-Curtis distances) revealed moderate grouping of the samples coming from different habitats. Forest and grassland samples were separated along the first nMDS axis, with wetland being intermediate **(Fig 3C)**.

**Fig 4:**
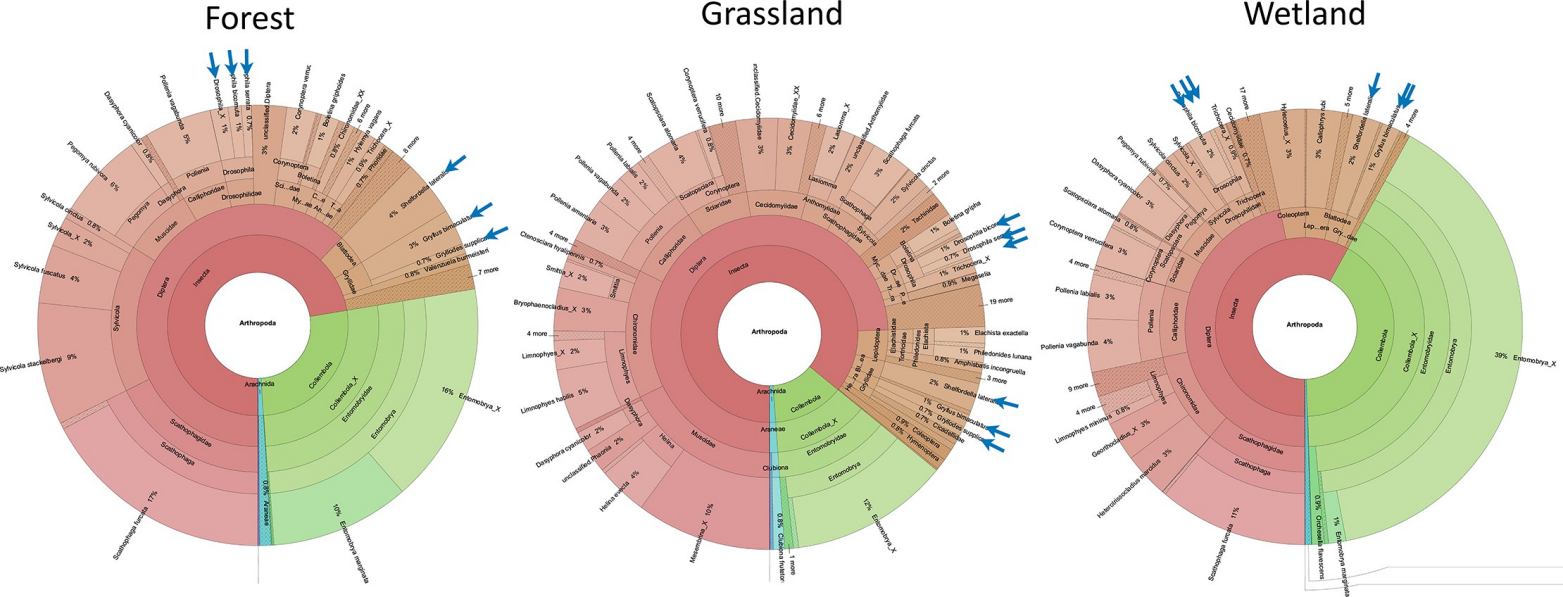
Taxonomic composition of samples collected in three different habitats. The Krona plots show the relative abundance of different taxa in a compound representation of five individual samples pooled per habitat. Blue arrows point to the spike-in species. Interactive versions of the Krona plots are available at GitHub: https://github.com/ela-iwaszkiewicz/Lab_protocols.git.

Modern data visualization tools for metabarcoding data, like Krona plots [44], allow users to visualize and compare the sample composition at different taxonomic levels **(Fig 4).** Note that the spike-in species—insects added to each sample before processing—are present in all habitats (blue arrows in **Fig 4**) and all samples. Their read abundances are substantial but do not take over, serving as a positive control of the protocol.

**Fig 5:**
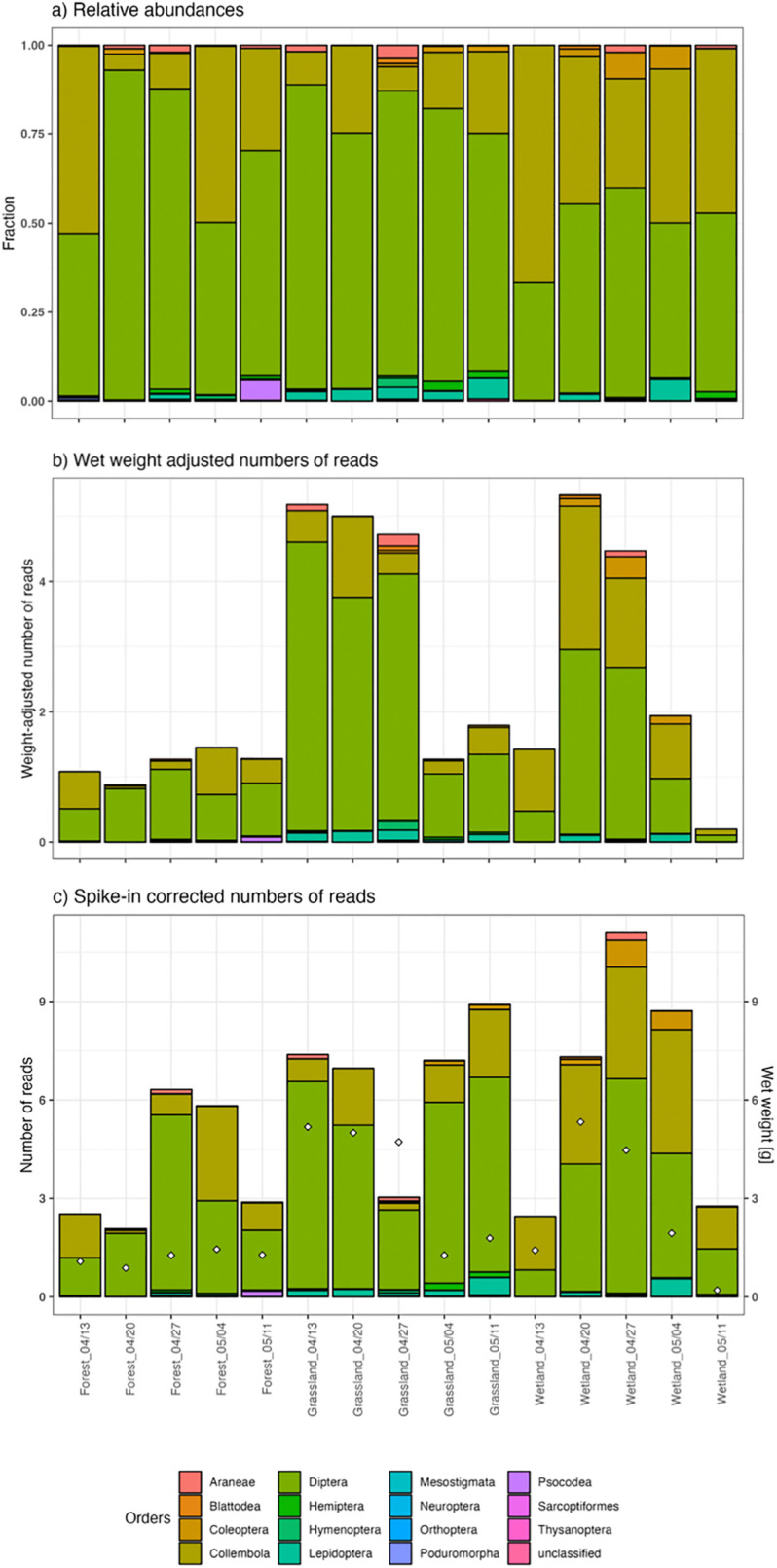
Abundance distributions of insect orders in the samples, using different normalization procedures. **a)** Relative abundance of ASVs grouped by their taxonomic assignment at the order level. Spike-in ASVs were excluded from the data. **b)** Relative abundance of reads adjusted to each sample’s biomass (wet-weight). Spike-in ASVs were excluded from the data. **c)** Spike-in corrected read counts. Colors represent arthropod orders and white diamonds identify the wet weight of the sample (scale shown on the right y-axis).

The traditional way of comparing community samples is based on relative abundance of taxa **(Fig 5A)**. However, areas of active investigation include the reconstruction and comparison of counts or biomass for different species present in the sample. The incorporation in the protocol of both the wet-weighing step and the biological spike-in insect additions provide us with two alternative ways of estimating abundances. First, we can take a look at the abundances of different orders per sample adjusted to the wet-weight of the sample **(Fig 5B)**. Alternatively, we can use the information from the spike-in insect reads to calibrate our counts table. We performed the corrections and standardization as suggested in Luo et al. 2023 [50] (summarized in their Fig 1, steps a-d). In other words, we adjusted the observed ASV counts table (i.e. the number of reads per ASV in each sample) by dividing each read count by the number of observed spike-in reads. These calculations were performed with reference to a weighted mean as calculated across all added spike-in specimens (e.g. mean[(*S*.lateralis*2 specimens) + (*G*.bimaculatus*1) + (*G*.sigilatus*1) + (*D*.serrata*1) + (*D*.jambolina*1) + (*D*.bicornuta*3)]) **(Fig 5C)**. It is important to note that adjusting read counts using wet weight or biological spike-ins can reduce the between-sample variation introduced during lab processing (referred to as “pipeline noise” by Luo et al. 2023 [50]) and allows for improved within-species quantitative comparison across samples, but it does not correct for species-specific biases such as differing DNA yield, preferential PCR amplification etc.

## Conclusions

Novel DNA-based methods have the potential to revolutionize biodiversity discovery and monitoring when applied in a high-throughput fashion. Swift processing is crucial for monitoring purposes as well as for informed decision making in conservation efforts.

The metabarcoding protocol described here allows a trained lab technician to process 180 samples (2 x 96-well plates when we include all negative and positive controls), from bulk insect catches to ready-to-sequence libraries, in 7 working days, translating to over 500 Malaise trap samples processed per month (for details see S1 Table). When processing samples at a scale of thousands, an estimated average per-sample reagent cost amounts to about 5 EUR for DNA extraction and purification using homemade magnetic bead solution and 3 EUR for library preparation; additionally, the costs of generating ~1M paired-end reads (2 x 250bp) per sample was about 10 EUR when using NovaSeq 6000 SPrime flow cell. Costs presented here are average costs when implementing the protocol in a high throughput manner, making use of bulk purchase of reagents and consumables, and using home made magnetic beads instead of standardized kits for DNA purification (as described in alternative step 17 in the step-by-step protocol uploaded in protocols.io). Neither these consumables/services costs nor the amount of labor involved (and associated human resources costs) are trivial. However, for large projects addressing grand questions about the biodiversity patterns during times of global change, they are not implausible. Also, there is space for the improvement of time- and cost-efficiency through more extensive use of laboratory automation, or skipping or replacing labor-intensive steps such as agarose gel-based library quality control after both the first and the second PCR.

We have shown using a small subset of processed samples that using FAVIS results in good quality metabarcoding data that can be used in biodiversity studies and be subject to biological interpretation. While hard to demonstrate in a quantitative manner, we have invested substantial effort in addressing and controlling some of the known methodological challenges including cross-contamination during sample processing and through index hopping, both of which had a measurable effect in our early datasets. The non-destructive nature of the protocol and the retention of specimens post-digestion allows for their future individual characterization using sequencing- or morphology-based studies. At the same time, it is important to pinpoint some of the challenges, likely to become more significant as sample collection and processing accelerates. Among the most important is sample management and tracking. When processing 7000 bulk insect samples from the Insect Biome Atlas project using this protocol, we simplified and streamlined sample management and data recording through the use of QR codes for sample labeling and storage location that are read and registered into a database via a handheld barcode scanner. Another important challenge is the long-term storage of samples and lysates. Those processed as a part of the current project occupy a substantial portion of a custom-build freezer house; but the availability of infrastructure and long-term storage costs could hamper some projects. The third major consideration are the challenges in the analysis and biological interpretation of tremendous amounts of data generated by the project. The bioinformatic workflow presented here is suitable for the analysis of much larger datasets, but dedicated statistical, modeling, and visualization solutions are needed before we can understand the patterns.

## Supporting information

S1 Fig Original, uncropped electrophoresis gel picture underlying Fig 2A from the main text. It shows products of the PCR I—length of the product was ca. 490bp. Yellow frame indicates which parts of the gel were presented in the main text. The Gel was 2.5% agarose and the DNA ladder used was Perfect Ladder 100-1000bp (EurX, Poland).
(PNG)

S2 Fig Original, uncropped electrophoresis gel picture underlying Fig 2B from the main text. It shows products of the PCR II—length of the product was ca. 563bp. Yellow frame indicates which parts of the gel were presented in the main text. The Gel was 2.5% agarose and the DNA ladder used was Perfect Ladder 100-1000bp (EurX, Poland).
(PNG)

S1 Table Timeline for the processing of 184 samples using FAVIS protocol. (PDF)

S1 File FAVIS protocol downloaded from protocols.io. (PDF)

S2 File (PDF)
