# Supplementary figures and images for: FAVIS: Fast and versatile protocol for non-destructive metabarcoding of bulk insect samples
Source: PLoS One. 2023 Jul 19;18(7):e0286272. doi: 10.1371/journal.pone.0286272 (PMC10356154; doi:10.1371/journal.pone.0286272)

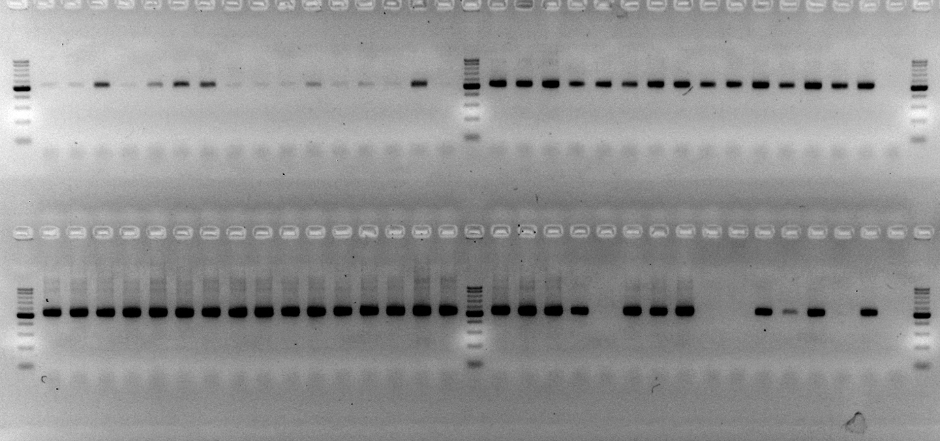

Supplement: S1 Fig — It shows products of the PCR I—length of the product was ca. 490bp. Yellow frame indicates which parts of the gel were presented in the main text. The Gel was 2.5% agarose and the DNA ladder used was Perfect Ladder 100-1000bp (EurX, Poland). (PNG) [file pone.0286272.s001.png]

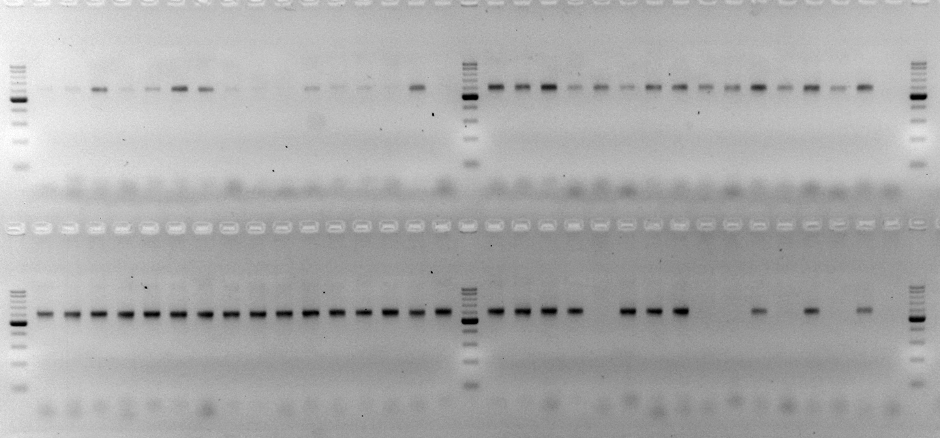

Supplement: S2 Fig — It shows products of the PCR II—length of the product was ca. 563bp. Yellow frame indicates which parts of the gel were presented in the main text. The Gel was 2.5% agarose and the DNA ladder used was Perfect Ladder 100-1000bp (EurX, Poland). (PNG) [file pone.0286272.s002.png]
