# Supplementary material for: FAVIS: Fast and versatile protocol for non-destructive metabarcoding of bulk insect samples
Source: PLoS One. 2023 Jul 19;18(7):e0286272. doi: 10.1371/journal.pone.0286272 (PMC10356154; doi:10.1371/journal.pone.0286272)
Supplement: S1 Table — (PDF) [file pone.0286272.s003.pdf]

Day	Sections	Steps		Duration (h)	Number of samples processed
day 1	section 1-2	step 1-15	Preparation and Sample processing (mild lysis)	8	40 samples + 1 negative control
day 2	section 1-2	step 1-15	Preparation and Sample processing (mild lysis)	8	40 samples + 1 negative control
day 3	section 1-2	step 1-15	Preparation and Sample processing (mild lysis)	8	40 samples + 1 negative control
day 4	section 1-2	step 1-15	Preparation and Sample processing (mild lysis)	8	40 samples + 1 negative control
day 5	section 1-2	step 1-15	Preparation and Sample processing (mild lysis)	6	19 samples + 1 negative control
day 5	section 3	step 16	DNA purification (using robot)	2	2x92 samples
day 6	section 4	step 18	Library preparation (PCR1 + PCR2)	8	2x92 samples
day 7	section 4	step 19	pooling*	8	2x92 samples

*If you use the same sequencing strategy as in FAVIS protocol you should only do this step when you have libraries corresponding to 8 plates. You can then create two Master pools, each composed of 4 plates.
