# Supplementary material for: FAVIS: Fast and versatile protocol for non-destructive metabarcoding of bulk insect samples
Source: PLoS One. 2023 Jul 19;18(7):e0286272. doi: 10.1371/journal.pone.0286272 (PMC10356154; doi:10.1371/journal.pone.0286272)
Supplement: S1 File — (PDF) [file pone.0286272.s004.pdf]

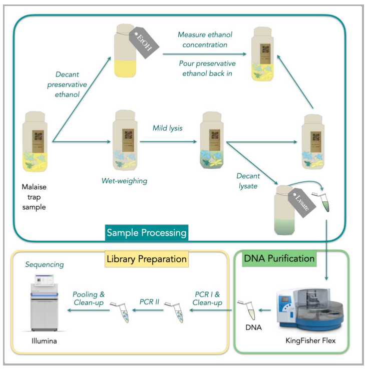

FAVIS: Fast and Versatile protocol for metabarcoding of bulk Insect Samples from large-scale diversity monitoring projects V.1

VERSION 1

MAY 15, 2023

Elżbieta Iwaszkiewicz-Eggebrecht[1], Piotr Łukasik[2], Mateusz Buczek[2], Junchen Deng[2,3], Emily Hartop[4,5], Harald Havnås[4], Monika Prus-Frankowska[2], Carina R. Ugarph[4], Paulina Viteri[1], Anders F. Andresson[6], Tomas Roslin[7], Ayco J. M. Tack[8], Fredrik Ronquist[1], Andreia Miraldo[1]

[1]Department of Bioinformatics and Genetics, Swedish Museum of Natural History, Box 50007, SE-104 05 Stockholm, Sweden;
[2]Institute of Environmental Sciences, Faculty of Biology, Jagiellonian University, ul. Gronostajowa 7, 30-387 Kraków, Poland;
[3]Doctoral School of Exact and Natural Sciences, Jagiellonian University, Kraków, Poland;
[4]Station Linné, Skogsby 161, 386 93 Färjestaden, Sweden;
[5]Center for Integrative Biodiversity Discovery, Museum für Naturkunde - Leibniz Institute for Evolution and Biodiversity Science, Invalidenstraße 43, 10115 Berlin, Germany;
[6]Science for Life Laboratory, Department of Gene Technology, KTH Royal Institute of Technology, 171 21 Stockholm, Sweden;
[7]Department of Ecology, Swedish University of Agricultural Sciences, Uppsala, Sweden;
[8]Department of Ecology, Environment and Plant Sciences, Stockholm University, Stockholm, Sweden

IBA

ela.iwaszkiewicz

OPEN ACCESS

DOI: dx.doi.org/10.17504/protocols.io.kqdg36261g25/v1

Protocol Citation: Elżbieta Iwaszkiewicz-Eggebrecht, Piotr Łukasik, Mateusz Buczek, Junchen Deng, Emily Hartop, Harald Havnås, Monika Prus-Frankowska, Carina R. Ugarph, Paulina Viteri, Anders F. Andresson, Tomas Roslin, Ayco J. M. Tack, Fredrik Ronquist, Andreia Miraldo 2023. FAVIS: Fast and Versatile protocol for metabarcoding of bulk Insect Samples from large-scale diversity monitoring projects. **protocols.io** https://dx.doi.org/10.17504/protocols.io.kqdg36261g25/v1 Version created by ela.iwaszkiewicz

License: This is an open access protocol distributed under the terms of the Creative Commons Attribution License, which permits unrestricted use, distribution, and reproduction in any medium, provided the original author and source are credited

Protocol status: Working
We used this protocol for more than 7000 Malaise trap samples and it's definitely working.

Created: Apr 18, 2023

Last Modified: May 15, 2023

PROTOCOL integer ID: 80701

Keywords: Metabarcoding, non-destructive lysis, high-throughput, insect diversity, Malaise trap, monitoring

ABSTRACT

Here we describe a non-destructive metabarcoding protocol that is optimized for high-throughput processing of Malaise trap samples and other bulk insect samples. The protocol details the process from obtaining bulk samples up to submitting them for sequencing. It is divided into four sections: 1) Laboratory workspace preparation; 2) Sample processing - decanting ethanol, measuring the wet-weight biomass and the concentration of the preservative ethanol, performing non-destructive lysis and preserving the insect material for future work; 3) DNA extraction and purification; and 4) Library preparation and sequencing. The protocol is cost-effective and relies on readily available reagents and materials. For the steps that require expensive infrastructure – such as the DNA purification robots or sequencing center services – we suggest alternative low-cost solutions when possible. The use of this protocol yields a comprehensive description of the number of species present in a given sample, their relative read abundances and the overall insect biomass. To date, we have successfully applied the protocol to more than 7000 Malaise trap samples obtained from Sweden and Madagascar. The protocol allows one lab technician to process 180 samples (2x96-well plates when we include all negative and positive controls), from bulk insect catches to ready-to-sequence libraries, in 7 working days. In other words, samples collected over 1 week from 565 Malaise traps can be processed in one month, allowing the timely delivery of the results.

GUIDELINES

Method for wet-weighing of insect biomass from Malaise trap samples
Two different methods to calibrate the timing of ethanol drainage from Malaise trap samples are described, including 1) dripping interval between ethanol drops and 2) time since the start of ethanol draining.
Methods
To establish a reliable method for wet-weighing of the biomass from Malaise trap samples, we used ten Malaise trap samples with visually different amounts of insect biomass, each filled with 450ml of 95% ethanol. These samples were used to test two methods of ethanol draining before wet-weighing the biomass: The first method (method 1) used different dripping time intervals before wet-weighing, as adapted from Hallmann et al. (2017), while the second method (method 2) additionally used time since the start of ethanol draining. To use the same samples for testing both methods, samples were refilled with 450 ml 95% ethanol after testing the first method. All samples were prepared for ethanol draining in the same way.
To prepare samples for ethanol draining, samples were carefully opened and a pre-cut nylon mesh circle (7 mm diameter, 300 µM aperture) was placed over the bottle opening. A plastic bottle seal was placed over the mesh to tighten it, and to keep it centred during draining. For one sample at a time, the ethanol from the insect bottle was drained into another bottle (the ethanol receiving bottle) using a funnel by carefully tilting the sample and rotating it until most of the ethanol was drained from the insect bottle and the ethanol started dripping. Once ethanol started dripping

from the bottle we placed the bottle against the funnel walls at an angle of 45° . We set a chronometer at the exact time the ethanol started flowing from the sample. During draining, we measured the absolute weight of the sample (i.e. weight of the insect biomass, bottle, nylon mesh and bottle seal) at several occasions. For method 1, we measured the absolute weight of the sample when the time between two drops falling from the bottle was 1, 2, 5, 10, 20 and 50 seconds. For method 2, the absolute weight of the samples was measured after 1, 5, 10, 15, 20, 25, 30, 40, 45 and 60 minutes since the start of ethanol draining. Additionally, the absolute weight of all samples was measured after leaving the sample overnight without a lid for 16 hours at room temperature. The wet-weight of the insect biomass was calculated by subtracting the weight of the bottle, nylon mesh and bottle seal from the absolute weight.

Results

For method 1, wet-weights of insect biomass considerably dropped from the start of ethanol draining to a dripping-interval of 10 seconds, especially so for larger samples (fig. S1.1a, table S1.1a). However, once the dripping-interval reached 20 seconds, the wet-weight of insect biomass stabilized, irrespective of the starting weight of the sample (fig. S1.1a, table S1.1a). Samples that were left overnight lost on average 2.9 grams of wet weight compared to samples with a dripping-interval of 50 seconds.

For method 2, wet-weights of insect biomass considerably dropped during the first 10 minutes since the start of ethanol draining (fig. S1.1b, table S1.1b). After 20 minutes of draining, wet-weights stabilized for all samples irrespective of their initial weight (fig. S1.1b, table S1.1b). Samples that were left overnight lost on average 1.6 grams of wet weight compared to samples that were drained for 60 minutes.

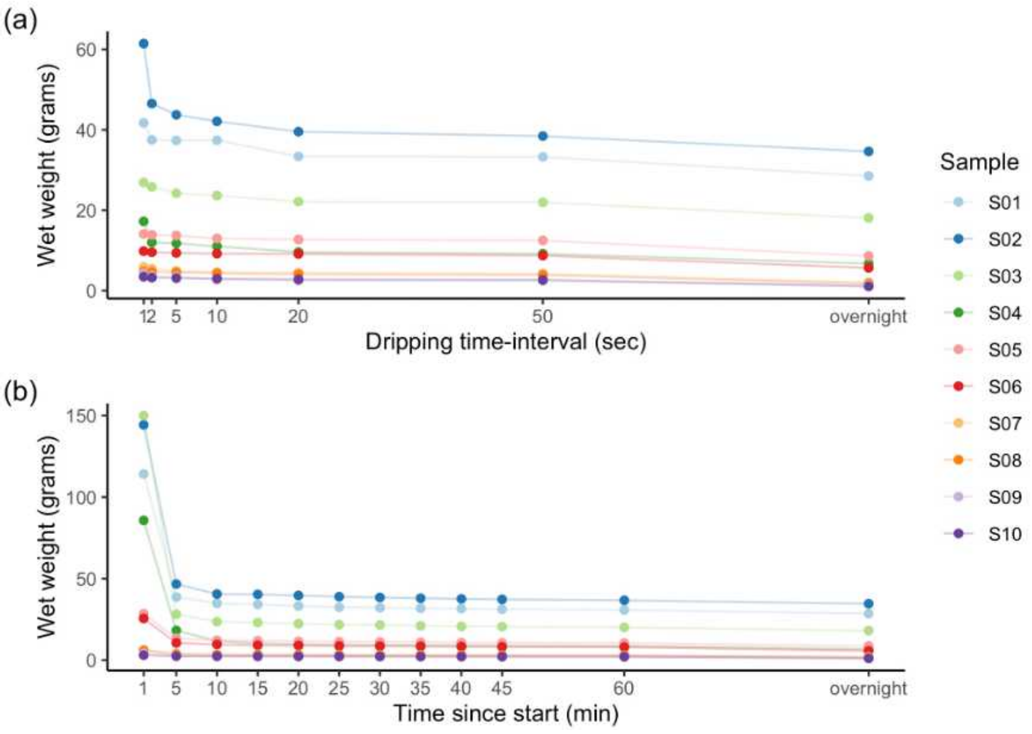

Figure S1.1. *Insect biomass from ten samples after ethanol drainage following (a) method 1 and (b) method 2. Panel (a) shows the wet weights of samples with a dripping interval of 1, 2, 5, 10, 20 and 50 seconds (method 1). Panel (b) shows the wet weight of samples that were drained for 1, 5, 10, 15, 20, 25, 30, 35, 40, 45, and 60 minutes (method 2). The wet weight of samples that were left overnight (16 hours) is shown in both panels. For the raw data on wet weight measurements, see table S1.1.*

Table S1.1. Insect biomass from ten samples after ethanol drainage following **(a)** method 1 and **(b)** method 2. Panel **(a)** shows the measurements of wet weights of samples (in grams) with a dripping interval of 1, 2, 5, 10, 20 and 50 seconds (method 1), as well as the wet weight of the samples after overnight drying (16 hours). Panel **(b)** shows the wet weight of samples (in grams) that were drained for 1, 5, 10, 15, 20, 25, 30, 35, 40, 45, and 60 minutes (method 2), as well as the wet weight of the samples after overnight drying (16 hours).

(a) Method 1

A	B	C	D	E	F	G	H
Sample	*Dripping interval*						overnight
	1 sec	*2 sec*	*5 sec*	*10 sec*	*20 sec*	*50 sec*	
S01	*41.71*	*37.50*	*37.37*	*37.42*	*33.37*	*33.26*	*28.52*

A	B	C	D	E	F	G	H
S02	*61.45*	*46.54*	*43.72*	*42.11*	*39.54*	*38.43*	*34.62*
S03	*26.89*	*25.79*	*24.23*	*23.64*	*22.13*	*21.96*	*18.06*
S04	*17.22*	*11.98*	*11.76*	*11.02*	*9.59*	*9.18*	*6.72*
S05	*14.09*	*13.88*	*13.72*	*12.98*	*12.71*	*12.48*	*8.63*
S06	*9.81*	*9.54*	*9.37*	*9.20*	*9.13*	*8.72*	*5.66*
S07	*5.93*	*5.43*	*4.92*	*4.61*	*4.50*	*4.24*	*2.05*
S08	*4.78*	*4.65*	*4.55*	*4.30*	*4.06*	*3.90*	*1.72*
S09	*4.28*	*3.65*	*3.48*	*2.75*	*2.53*	*2.40*	*1.36*
S10	*3.45*	*3.24*	*3.10*	*2.97*	*2.79*	*2.70*	*1.05*

(b) Method 2

A	B	C	D	E	F	G	H	I	J	K	L	M
Sample	*Time since start of draining*											over night
	1 min	*5 min*	*10 min*	*15 min*	*20 min*	*25 min*	*30 min*	*35 min*	*40 min*	*45 min*	*60 min*	
S01	*114.23*	*38.68*	*34.81*	*34.16*	*33.19*	*32.42*	*32.16*	*31.81*	*31.54*	*31.22*	*30.81*	*28.52*
S02	*144.32*	*46.66*	*40.52*	*40.30*	*39.66*	*38.91*	*38.45*	*37.98*	*37.57*	*37.27*	*36.70*	*34.62*
S03	*150.03*	*28.08*	*23.56*	*23.04*	*22.33*	*21.74*	*21.61*	*21.06*	*20.71*	*20.52*	*20.11*	*18.06*
S04	*85.71*	*18.18*	*11.61*	*10.32*	*9.76*	*9.48*	*9.36*	*9.14*	*8.99*	*8.85*	*8.69*	*6.72*
S05	*28.48*	*13.38*	*12.17*	*11.91*	*11.54*	*11.32*	*11.16*	*10.94*	*10.78*	*10.65*	*10.47*	*8.63*
S06	*25.55*	*10.58*	*9.59*	*9.05*	*8.92*	*8.66*	*8.55*	*8.44*	*8.29*	*8.16*	*7.98*	*5.66*
S07	*6.00*	*4.08*	*3.64*	*3.52*	*3.42*	*3.34*	*3.28*	*3.22*	*3.16*	*3.10*	*3.04*	*2.05*
S08	*6.15*	*3.54*	*3.32*	*3.26*	*3.21*	*3.16*	*3.11*	*3.06*	*3.02*	*2.98*	*2.92*	*1.72*

A	B	C	D	E	F	G	H	I	J	K	L	M
S09	*4.15*	*2.62*	*2.49*	*2.43*	*2.38*	*2.34*	*2.30*	*2.26*	*2.22*	*2.18*	*2.13*	*1.36*
S10	*3.05*	*2.41*	*2.35*	*2.31*	*2.27*	*2.23*	*2.20*	*2.18*	*2.14*	*2.10*	*2.06*	*1.05*

References:

Hallmann CA, Sorg M, Jongejans E, Siepel H, Hofland N, et al. (2017)More than 75 percent decline over 27 years in total flying insect biomass in protected areas. PLOS ONE 12(10): e0185809. https://doi.org/10.1371/journal.pone.0185809

MATERIALS

In this section we list consumables, chemicals and reagents that are needed to run the protocol. This is not an exhaustive list as we assume that you have access to common lab equipment (pipettes, centrifuges, vortex, etc) and certain common lab consumables (filtered pipette tips, petri dishes etc.). For less commonly used lab equipment and consumables that are used in the protocol we opt to reference them below.

LIST OF EQUIPMENT

A	B	C
Item	Brand	Reference
Bottle seal (x40)	homemade	
Water bath	homemade	
Plastic container, 80 Litre (x 2)		
Adjustable 8-channel pipette (15-1250 uL)	Thermo Scientific™	4672090BT
Density meter instrument	Anton Paar	172244
Ethanol retrieving bottle 500 mL (x40)	Fisher scientific	2189-0016
Funnel (x40)	BRAND™	148040
Large forceps, 200 mm long (x40)	VWR®	232-2196
Beaker, 10 mL (x40)	BRAND™	91212
Shaking incubator - INCU-Line ILS 6	VWR®	444-0763
Small tweezers, 110 mm long (x5)	Sargenta	802-50
Tray (x40)	Buerkle™	4201-1318
KingFisher Flex robot	Thermo Scientific™	5400620

A	B	C
Qubit fluorometer instrument	Invitrogen™	Q33238
Magnetic stand for 1.5 mL microtubes	Invitrogen™	12321D
Magnetic stand for 96-well plates (x2, one for pre-PCR room and one for pos-PCR room)	Invitrogen™	AM10027
Plate Shaker, (x2, one for pre-PCR room and one for pos PCR room)	Thermo Scientific	50094308
Precision scales		
Squirt bottle (x2, one for bleach solution and one for 95% EtOH)		
Graduated cylinders (50 mL, 100 mL, 200 mL, 300 mL, 400 mL)		

LIST OF CONSUMABLES

A	B	C
Item	Brand	Reference
Nalgene 500 ml collecting bottle (insect bottle)	Fisher scientific	2105-0016
Nylon mesh circle - Ø7 cm, 300 uM aperture (2 per sample)	Megaview - bugdorm	BDZ0001
Ethanol retrieving bottle (500 ml capacity, wide mouth)	Fisher scientific	2189-0016
Lysate collecting bottle 250 mL	Fisher scientific	2189-0008
Lysate collecting bottle 500 mL	Fisher scientific	2189-0016
96-well microtube rack	VWR®	211-0213
96 deep-well 96 plate (2.2 mL)	Thermo Scientific™	95040450
Reagent reservoirs (100 mL)	Thermo Scientific™	95128085
96-well plate (0.5 mL)		
Colour stickers (5 different colours)		
Lysate microtubes (2 mL screw cap)		
Falcon tube (50 mL)		
Microtubes (1.5 mL)		

CHEMICALS AND OTHER REAGENTS

- Sodium hypochloride solution (NaOCl) 47/50% - CAS: 7681-52-9
- Sodium dodecyl sulphate (SDS) - CAS: 151-21-3
- Sodium Chloride (NaCl) - CAS: 7647-14-5
- Tris ultrapure 99,9% - CAS: 77-86-1
- Hydrochloric acid (HCl) - CAS: 7647-01-0
- EDTA disodium dihydrate - CAS: 6381-92-6

- PEG-8000 - CAS: 25322-68-3
- Tween 20 - CAS: 9005-64-5
- Ethanol 99.9% (EtOH)
- Carboxyl-modified Sera-Mag Magnetic Speed-Beads (Hydrophobic) (Cytiva, cat. #65152105050250)
- Proteinase K Solution (20 mg/mL) - OMEGA, ref.PROK50
- KingFisher™ Cell and Tissue DNA Kit - THERMO SCIENTIFIC™, discontinued
- Qiagen multiplex PCR mix - QIAGEN, ref.206145
- Qubit dsDNA HS assay kit - INVITROGEN, ref.Q32851
- Promega ProNex® Size-Selective Purification System ???????????????????
- DNase/RNase-Free Distilled Water (ddH2O)
- 100 µM CO1 primer BF3_P5_ins0 (see Primer info below for sequence details)
- 100 µM CO1 primer BF3_P5_ins1 (see Primer info below for sequence details)
- 100 µM CO1 primer BF3_P5_ins2 (see Primer info below for sequence details)
- 100 µM CO1 primer BF3_P5_ins3 (see Primer info below for sequence details)
- 100 µM CO1 primer BR2_P5_ins0 (see Primer info below for sequence details)
- 100 µM CO1 primer BR2_P5_ins1 (see Primer info below for sequence details)
- 100 µM CO1 primer BR2_P5_ins2 (see Primer info below for sequence details)
- 100 µM CO1 primer BR2_P5_ins3 (see Primer info below for sequence details)
- 100 µM i5 index (see Primer info below for sequence details)
- 100 µM i7 index (see Primer info below for sequence details)

STOCK SOLUTIONS AND BUFFERS

Below we describe recipes for all stock solutions and buffers used in the protocol. These should be prepared before starting the protocol.

LYSIS BUFFER, 5 L

We use the buffer by Vesterinen *et al.* (2016), who modified it from Aljanabi and Martinez (1997).

- Add 4.5 L of ddH20 to a 5 litre glass bottle.
- Add 116.9 g of Sodium Chloride (NaCl).
- Add 2.9 g EDTA disodium dihydrate.
- Add 50 mL 1M Tris-HCl, pH 8.0.
- Add 20 g of Sodium dodecyl sulphate (SDS).
- Dissolve the reagents by adding a magnetic flea stirrer into the glass bottle and placing the bottle on a magnetic stirrer at 60ºC. Close the bottle with the lid but make sure to leave the lid a bit loose.
- After all ingredients have dissolved, remove the magnetic stirrer from the bottle with the help of a metal magnet.
- Top up the solution to 5 L with ddH2O and firmly close the bottle with the lid.

5M NACL, 1 L

- Add 292 g of sodium chloride (NaCl) to a beaker.
- Adjust volume to 800 mL with ddH2O.
- Dissolve the sodium chloride by adding a magnetic flea into the beaker and placing on a magnetic stirrer.
- Top up the solution to 1 L with ddH2O.

- Sterilize by filtering the solution with a 0.2 uM filter membrane and store at room temperature.

1M TRIS-HCL, pH 8.0, 1 L

- Add 121.14 g Tris ultrapure 99.9% to a beaker.
- Adjust volume to 800 mL with ddH2O.
- Adjust pH to 8.0 with hydrochloric acid (HCl).
- Adjust volume to 1 L with ddH2O.
- Sterilize by filtering the solution with a 0.2 uM filter membrane and store at room temperature.

0.5M EDTA, pH 8.0, 1 L

- Add 186.12 g EDTA disodium salt to a beaker.
- Adjust volume to 1 L with ddH2O.
- Adjust pH to 8 with sodium hydroxide (NaOH).
- Sterilize by filtering and store at room temperature.

TE BUFFER, 1 L

- Add 100 mL 1M Tris-HCl, pH 8.0 to a 1 Litre glass bottle.
- Add 20 mL 0.5M EDTA, pH 8.0 to the bottle.
- Top up the solution to 1 L by adding 880 mL of ddH2O.
- Place the lid on the bottle and invert a few times to mix.

MAGNETIC BEAD SOLUTION, 50 mL

As an alternative to commercial kits, you can use home-made magnetic beads to purify the DNA. The homemade magnetic beads can be prepared according to Rohland and Reich (2012) by combining Carboxyl-modified Sera-Mag Magnetic Speed-Beads (Hydrophobic) (Cytiva, cat. #65152105050250) with polyethylene glycol (PEG)/NaCl buffer. The Sera-mag Speed-Beads contain sodium azide, so they first need to be washed twice with TE as described in steps 1-8 below.

1. Gently vortex Sera-mag Magnetic SpeedBeads and transfer 1 mL of beads to a 1.5 mL microtube.
2. Place the microtube tube on a magnetic stand until beads are drawn to magnet.
3. Remove the supernatant with P200 or P1000 pipette.
4. Add 1 mL TE buffer to the beads, remove from magnetic stand, mix thoroughly and return to magnet.
5. Remove supernatant with P200 or P1000 pipette.
6. Repeat steps 4-5.
7. Add 1 mL TE buffer to the beads and remove from the magnetic stand.
8. Fully re-suspend the beads by pipetting up and down. To be used in step 16 below.
9. Add 9 g PEG-8000 to a new 50 mL sterile Falcon tube.
10. Add 10 mL 5 M NaCL.
11. Add 500 µL 1 M Tris-HCL.
12. Add 100 µL 0.5 M EDTA.
13. Fill the Falcon to ~ 49 mL using sterile ddH20.
14. Gently mix the Falcon tube for about 5 minutes until you obtain a clear solution (PEG is completely dissolved into the solution).

15. Add 27.5 µL Tween 20 and mix gently.
16. Mix 1 mL of washed magnetic bead + TE solution prepared in step 8 above and transfer to the 50 mL Falcon tube.
17. If needed, top up the Falcon tube to 50 mL with ddH20.
18. Gently mix the Falcon tube until you obtain an homogeneous brown solution.
19. Wrap in tinfoil and store at 4°C.
20. Take out the magnetic beads solution from the fridge and keep at room temperature for 1 hour before using.
21. Vortex the magnetic beads solution thoroughly before use.

10 µM CO1_BF3_P5 PRIMER MIX, 1 mL

- Add 25 µL of 100 µM stock solution of each one of the 4 variable length forward primers (BF3_P5_ins0, BF3_P5_ins1, BF3_P5_ins2, BF3_P5_ins3) to a 1.5 mL microtube.
- Add 900 µL of TE buffer.

10 µM CO1_BR2_P5 PRIMER MIX, 1 mL

- Add 25 µL of 100 µM stock solution of each one of the 4 variable length reverse primers (BR2_P5_ins0, BR2_P5_ins1, BR2_P5_ins2, BR2_P5_ins3) to a 1.5 mL microtube.
- Add 900 µL of TE buffer.

PRIMER INFO FOR PCR1 and PCR2:

Amplicon libraries were prepared following a two-step PCR library preparation protocol (Method 4, Glenn et al., 2019a). In the first PCR, the 458-bp target region was amplified using template-specific primers with variable-length inserts and Illumina adapter tails. After the bead-purification of products, libraries were completed with Illumina adapters unique for each library in the second, indexing PCR. The overall library preparation strategy is presented in Figure X below. The following technical note https://ngisweden.scilifelab.se/2021/01/tech-note-increase-complexity-of-amplicon-libraries-using-phased-primers/ (Bonath F., 2021) describes in more detail the concept and advantages of the solutions used. Oligonucleotides were developed following Adapterama scheme (Glenn et al. 2019b).

For PCR1, we developed fusion primers based on broad-spectrum CO1 primers BR2 and BF3 primers (Elbrecht et al., 2019) modified with variable length inserts - 4 variants forward and 4 variants reverse. The primers are composed of template-specific primer (*italic*), variable length insert (bold) and a tail representing partial Illumina adapter sequence (unformatted):

forward

BF3_P5_ins0:

ACACTCTTTCCCTACACGACGCTCTTCCGATCT*CCHGAYATRGCHTTYCCHCG*

BF3_P5_ins1:

ACACTCTTTCCCTACACGACGCTCTTCCGATCT**A***CCHGAYATRGCHTTYCCHCG*

BF3_P5_ins2:

ACACTCTTTCCCTACACGACGCTCTTCCGATCT**GA***CCHGAYATRGCHTTYCCHCG*

BF3_P5_ins3:

ACACTCTTTCCCTACACGACGCTCTTCCGATCT**TGA***CCHGAYATRGCHTTYCCHCG*
reverse
BR2_P7_ins0:
GTGACTGGAGTTCAGACGTGTGCTCTTCCGATCT*CDGGRTGNCCRAARAAYCA*
BR2_P7_ins1:
GTGACTGGAGTTCAGACGTGTGCTCTTCCGATCT**T***CDGGRTGNCCRAARAAYCA*
BR2_P7_ins2:
GTGACTGGAGTTCAGACGTGTGCTCTTCCGATCT**AT***CDGGRTGNCCRAARAAYCA*
BR2_P7_ins3:
GTGACTGGAGTTCAGACGTGTGCTCTTCCGATCT**GAT***CDGGRTGNCCRAARAAYCA*
For PCR2, we developed primers combining Adapterama sequences with 10-nucleotide indexes, using "IDT for Illumina Nextera DNA UD Indexes" from "Illumina Adapter Sequences document #1000000002694 v11 (April 2019)" (marked as x´s below). The **bold** portion of the indexing primer overlaps with the PCR 1 product:
index i5 (forward):
AATGATACGGCGACCACCGAGATCTACACxxxxxxxxxx**ACACTCTTTCCCTAC**
index i7 (reverse):
CAAGCAGAAGACGGCATACGAGATxxxxxxxxxx**GTGACTGGAGTTCAG**
Below you can see a schematic representation on how these primer combinations work.

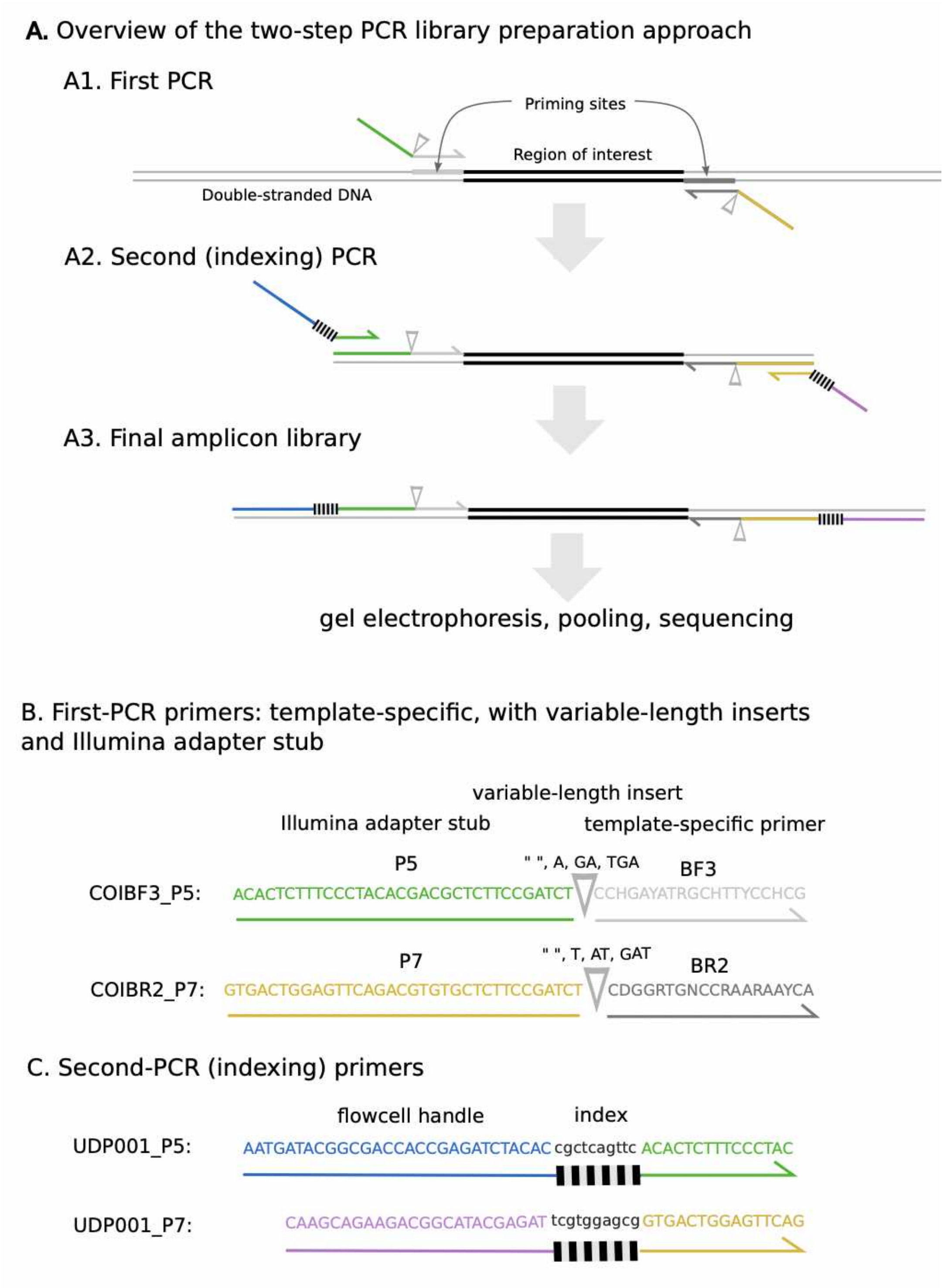

REFERENCES:

- Vesterinen, E.J., Ruokolainen, L., Wahlberg, N., Peña, C., Roslin, T., Laine, V.N., Vasko, V., Sääksjärvi, I.E., Norrdahl, K., Lilley, T.M., 2016. What you need is what you eat? Prey selection by the bat Myotis daubentonii. Mol. Ecol. 25, 1581–1594.https://doi.org/10.1111/mec.13564
- Aljanabi, S.M., Martinez, I., 1997. Universal and rapid salt-extraction of high quality genomic DNA for PCR-based techniques. Nucleic Acids Res. 25, 4692–

4693.https://doi.org/10.1093/nar/25.22.4692

- Rohland N, Reich D. 2012. *Cost-effective, high-throughput DNA sequencing libraries for multiplexed target capture.* Genome Research 22: 939-946. http://www.genome.org/cgi/doi/10.1101/gr.128124.111.
- Elbrecht V, Braukmann TWA, Ivanova NV, Prosser SWJ, Hajibabaei M, Wright M, Zakharov EV, Hebert PDN, Steinke D.2019.Validation of COI metabarcoding primers for terrestrial arthropods. PeerJ7:e7745. https://doi.org/10.7717/peerj.7745
- Glenn TC, Pierson TW, Bayona-Vásquez NJ, Kieran TJ, Hoffberg SL, Thomas IV JC, Lefever DE, Finger JW, Gao B, Bian X, Louha S, Kolli RT, Bentley KE, Rushmore J, Wong K, Shaw TI, Rothrock Jr MJ, McKee AM, Guo TL, Mauricio R, Molina M, Cummings BS, Lash LH, Lu K, Gilbert GS, Hubbell SP, Faircloth BC.2019.Adapterama II: universal amplicon sequencing on Illumina platforms (TaggiMatrix). PeerJ7:e7786. https://doi.org/10.7717/peerj.7786
- Glenn TC, Nilsen RA, Kieran TJ, Sanders JG, Bayona-Vásquez NJ, Finger JW, Pierson TW, Bentley KE, Hoffberg SL, Louha S, Garcia-De Leon FJ, del Rio Portilla MA, Reed KD, Anderson JL, Meece JK, Aggrey SE, Rekaya R, Alabady M, Belanger M, Winker K, Faircloth BC.2019.Adapterama I: universal stubs and primers for 384 unique dual-indexed or 147,456 combinatorially-indexed Illumina libraries (iTru & iNext). PeerJ7:e7755. https://doi.org/10.7717/peerj.7755

BEFORE START INSTRUCTIONS

SECTION 1: Preparation

1 **Set up washing stations**

1.1 Set up a 80 L plastic container with washing liquid and water.

1.2 Set up a 80 L plastic container with 10% bleach solution by adding nine parts water to one part laboratory bleach (NaOCl - sodium hypochlorite solution 47/50%).

NOTE 1: The size of the plastic containers will depend on the amount of samples you are processing. For the 41 samples, we use a plastic container of 80 litres capacity to allow washing all equipment used in one day.

NOTE 2: All equipment should be washed by first placing it in the 10% bleach container for at least 15 minutes, and then moved into the soap and water container to get rid of the bleach and for rinsing. Be aware that metal will corrode if placed longer than 10 minutes in bleach 10% solution.

NOTE 3: Empty the bleach and water and soap tanks weekly.

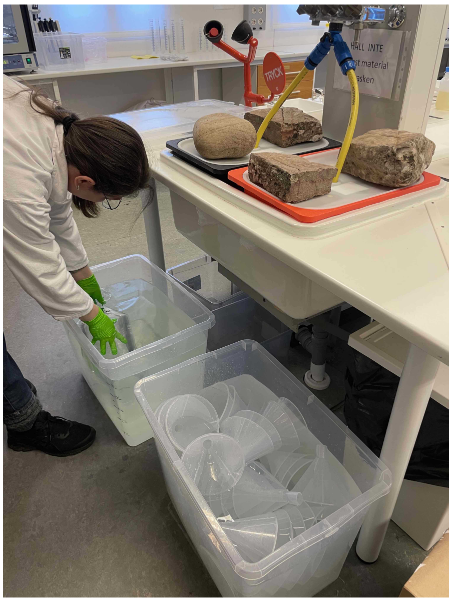

2 **Set up working stations**

This protocol is optimized to process 40 samples per day, plus one blank sample. Start by creating 41 individual working stations for each insect bottle and blank sample by dividing the lab bench into 30 cm wide working stations. Number each station from 1 to 41.

2.1 Use a squirt bottle with bleach 10% solution and a paper towel to wipe all lab benches.

2.2 At each working station place the following:

- 1 tray
- 1 ethanol retrieving bottle (500 mL capacity, wide mouth)
- 1 funnel (to place on top of the ethanol retrieving bottle)
- 1 bottle seal
- 2 nylon mesh circles Ø7 cm (one to decant ethanol in step 7.2 and one to decant lysate in step 14.3)
- 1 set of large forceps (200 mm long)

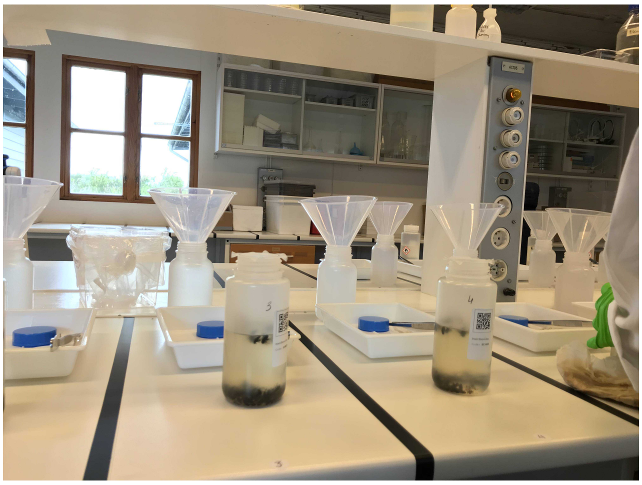

2.3 Place one insect bottle in each working station 1 to 40 and one empty bottle (Nalgene, 500 ml HDPE wide mouth) in working station 41 (blank sample). The blank sample should be labeled as B-DDMMYY (DD=day, MM=month, YY=year).

2.4 Using a marker pen, label each sample bottle and lid with the respective working station number (from 1 to 41).

3 **Set up spike-in stations**

This step can be skipped if you are not adding biological spike-ins to your samples. If you are adding spike-in species it is important to use species that are very unlikely to be naturally present in the surveyed community, and that are available as large numbers of genetically homogeneous, quality- and age-standardized specimens, whether from biocontrol providers, laboratory cultures, or other sources. Reserve a clean bench to set up a spike-in station as follows:

3.1 Place a petri dish and an individual set of forceps for each spike-in species you will be using.

3.2 Remove the spike-in species from the freezer and put a small amount of each spike-in species into their respective petri dish using one set of forceps for each species.

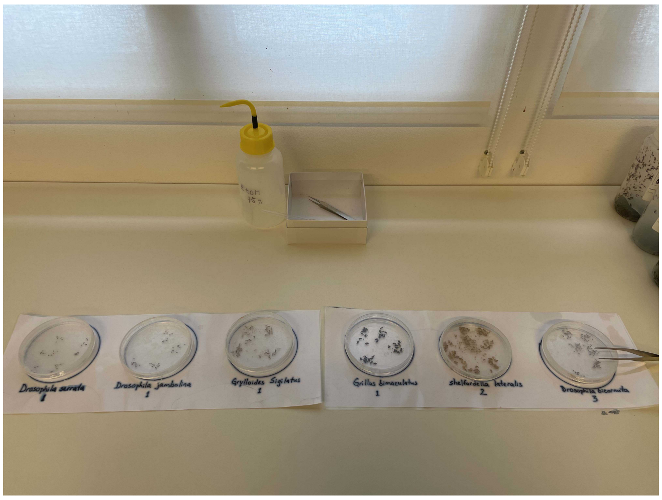

4 **Set up lysis buffer and proteinase K station**

Reserve a clean bench to set up a lysis buffer and proteinase K station. Make sure you have several sizes of graduated cylinders (50 to 500 mL) and small funnels so that you can add lysis buffer accurately to each insect bottle and pipettes and filter tips so that you can add the appropriate amount of proteinase K to each insect bottle. The amount of lysis buffer and proteinase K to be added to each sample range from 30 mL to 400 mL (buffer) and 30 µL to 400 µL (proteinase K) and depend on the insect biomass of each sample (see Table 2).

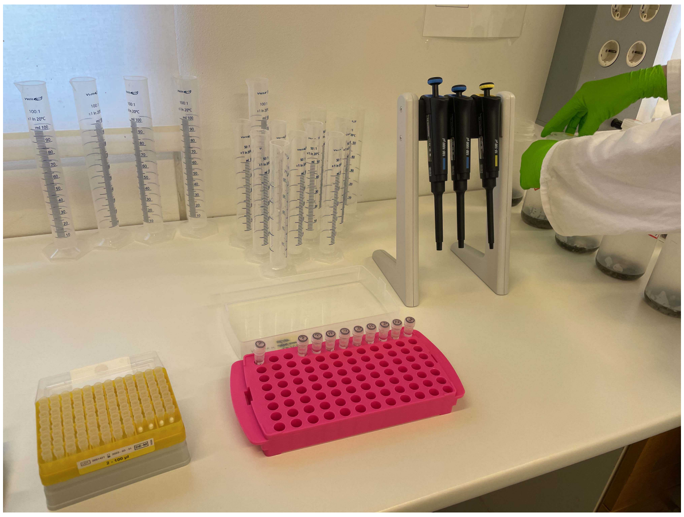

Lysis buffer and proteinase K station.

5 **Set up weighing station**

Reserve a clean bench to set up the weighing station. Make sure you have precision scales, five different coloured stickers (one color for each biomass category as shown in Table 1) and a laptop to register the weight of each sample.

NOTE 5: Please be aware that in step 8.2 we measure the absolute weight of each bottle with insects. To obtain the insect biomass we have to subtract from the absolute weight registered, the weight of the sample bottle, round mesh (that we add to the bulk insect community sample in step 7.9) and any other labels attached to the outside of the bottle with insects. Therefore, make sure you obtain the average weight of your insect collecting bottles, labels and mesh à priori so that you can calculate the insect biomass of your samples in step 8.3.

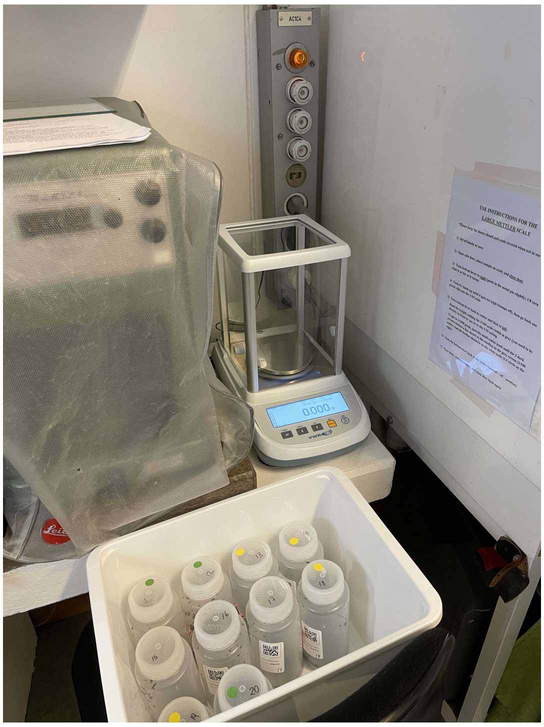

6 Preheat lysis buffer

Place bottles of lysis buffer (see Materials) into a large shaking incubator (we recommend INCU-Line ILS6; VWR) and set to 65-70°C, 90 rpm, to preheat. Invert the bottle every hour to help the salts fully dissolve - especially important during cold days as salts precipitate at low temperature.

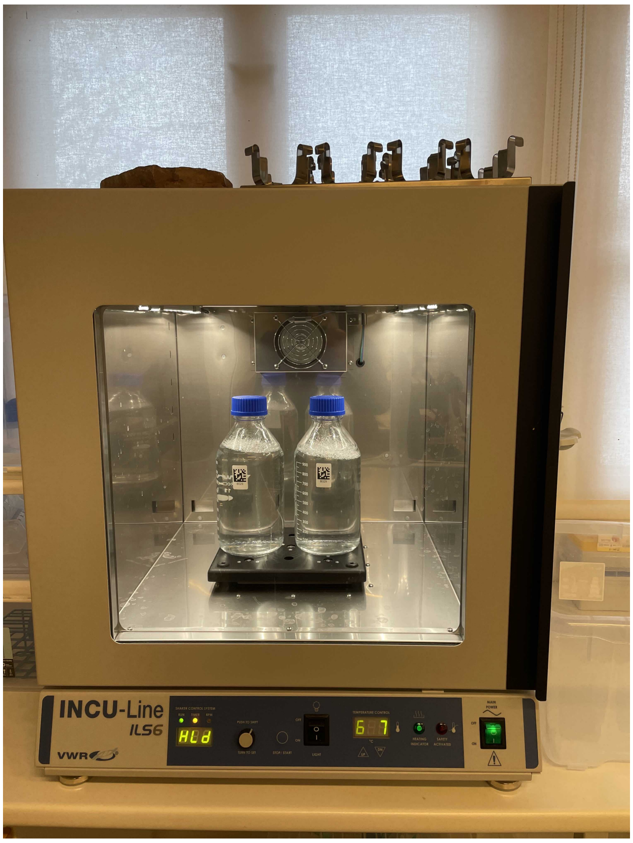

SECTION 2: Sample Processing

7 **Decant ethanol**

At the time of retrieving the insect bottle from the Malaise trap, we add an archive paper label inside the bottle where we write down all metadata associated with the sample (site/trap id, start and end date of collection and collector name). This archive label needs to be removed before decanting ethanol from the sample, as explained in steps 7.1 below. If you don´t have an archive paper label inside your bottles, then move on to step 7.2.

7.1 Starting in working station 1, open the insect bottle and take out the archive paper label (if you have one) with the large forceps. If necessary, use a squirt bottle with ethanol 95% to rinse down any specimens that are attached to the archive paper label so that they fall back inside the bottle.

7.2 Place one nylon mesh circle over the opening of the insect bottle and use the bottle seal to secure the mesh in place. Use the large forceps to help with this step.

7.3 Move to the next working station, and repeat steps 7.1-7.2 for each insect bottle.

7.4 Return to work station 1 and pour the preservative ethanol from the insect bottle slowly through the funnel and into the ethanol receiving bottle so that the majority of the ethanol is emptied from the insect bottle. Gently rotate the insect bottle as necessary to facilitate the draining of ethanol.

7.5 Turn the insect bottle upside down and let it rest in the funnel, so that the ethanol continues to drain.

7.6 Set a timer for 35 minutes after the first sample to ensure that most of the ethanol will be drained. See "Guidelines & Warnings" section where we describe why we use 35 minutes to get accurate measures of wet-weigh insect biomass.

7.7 Move to the next working station, and repeat steps 7.4-7.5 for each insect bottle.

7.8 After 35 minutes have passed, return to working station 1 and remove the insect bottle from the funnel.

7.9 Use the forceps to carefully remove the seal from the insect bottle and push the round mesh through, so that it falls to the bottom of the bottle.

7.10 Close the insect bottle with its original bottle cap.

7.11 Repeat steps 7.8-7.10 at all working stations.

7.12 Cycle through all stations and transfer the funnel from each station to the bleach container in the washing station and close the ethanol receiving bottle with its respective bottle cap.

8 Wet weigh samples

8.1 Move all insect bottles to the weighing station.

8.2 Weigh each insect bottle and enter the data into the dedicated spreadsheet.

8.3 To obtain the insect biomass, subtract the weight of the bottle+round mesh+any other labels attached to the bottle from the absolute weight measured in step 8.2 (see Note 5 in step 5 “Set up weighing station” above). We advise creating an excel file where you have a formula that automatically calculates the insect biomass from the absolute weight that you register. Also, by coloring the cell automatically according to the biomass category (see step 8.4 below) you immediately know which insect biomass category each sample falls into.

8.4 Sort weighed insect bottles into insect biomass categories by putting a color tag on the top of each bottle (see colour codes in Table 1).

A	B
Sample weight (g)	Colour tag
< 5.00	red
5.00 – 9.99	yellow
10.00 - 19.99	green
20.00 - 29.99	blue
30.00 - 39.99	black

A	B
> 40.00	white

Table 1. Colour tag to be added to each insect bottle according to the insect biomass.

8.5 Move all insect bottles to the spike-in station (if you are adding spike-ins) or directly to the lysis station (if you are not adding spike-ins).

9 **Add biological spike-ins (OPTIONAL)**

Working with one insect bottle at a time, remove the lid and add spike-in insects as below, then transfer the insect bottle and lid to the lysis station. Do not add spike ins to your blank sample!

Drosophila serrata – 1 specimen
Drosophila jambulina – 1 specimen
Drosophila bicornuta – 3 specimens
Shelfordella lateralis - 2 specimens
Gryllodes sigillatus - 1 specimen
Gryllus bimaculatus - 1 specimen

10 **Adding lysis buffer and proteinase K**

10.1 At the lysis station, add the appropriate amount of buffer to each insect bottle according to Table 2 using the graduated cylinders and a small funnel to get the right amount of buffer. The funnel is important so that the lysis buffer is poured slowly and accurately from the 1L bottle into the graduated cylinder, reducing foaming.

NOTE 6: When adding the lysis buffer to each insect bottle, use the buffer to rinse the insect bottle walls so that all specimens that are attached to the walls fall at the bottom of the bottle and are soaked in the buffer. See video below.

A	B	C
Sample weight (g)	Lysis buffer (mL)	Proteinase K (µL)
< 5.00	30	30
5.00 – 9.99	50	50
10.00 - 19.99	100	100
20.00 - 29.99	200	200
30.00 - 39.99	300	300
> 40.00	400	400
Blank sample	50	50

Table 2. Specification on amount of lysis buffer and proteinase K to be added to each insect bottle relative to the insect biomass of each sample.

10.2 Add the correct amount of proteinase K as shown in Table 2 to each insect bottle.

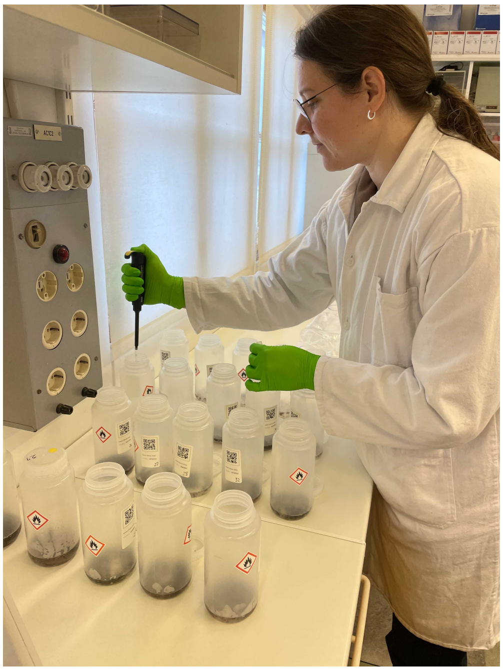

10.3 Close all insect bottles with their respective bottle caps.

NOTE 7: To speed up this step you can first add proteinase K to the lysis buffer before adding the mixed solution to the insect bottles. To minimize waste, first calculate how much buffer you will need for the samples you are processing that day (you will know this after having the wet-weight of each sample). Add the correct amount of proteinase K to the total amount of buffer and then simply add the correct amount of "lysis buffer + proteinase K" solution to each insect bottle according to Table 12.

11 **Preheat insect bottles in water bath**

It is important to preheat the insect bottles in a water bath before starting the incubation period as they take time to heat up to the desired incubation temperature of 56°C in a dry incubator. The water bath speeds up this process considerably and ensures that each sample is at 56°C when starting the incubation period in the dry incubator.

You can use a commercial shaking water bath or you can create your own one as we did in our lab (see figures below for an explanation). For small size samples (30 mL, 50 mL, 100 mL) it takes 30 minutes of pre-heating in the water bath for the samples to reach the desired temperature of 56°C, for large samples (200 mL, 300 mL, 400 mL) it will take 40 minutes. This timings are specific to the bottles we use to collect insects and the temperature of the tap water that circulates in the water bath (60°C in our case) and they might need to be adjusted for your own project.

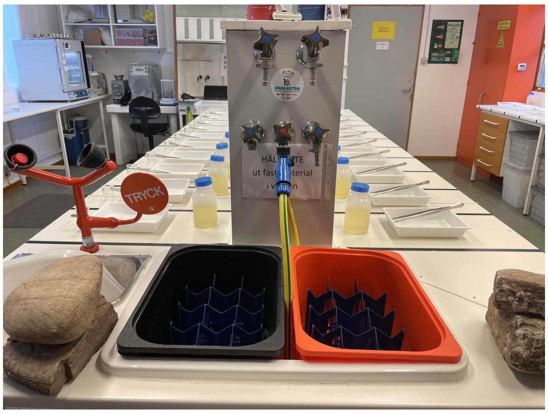

Home-made water bath system consists of two water containers (red and black in this photo), a tap with hot water with a double exit hose, bottle holders (inside the water containers), water container lids and weights (rocks) to keep the lids on.

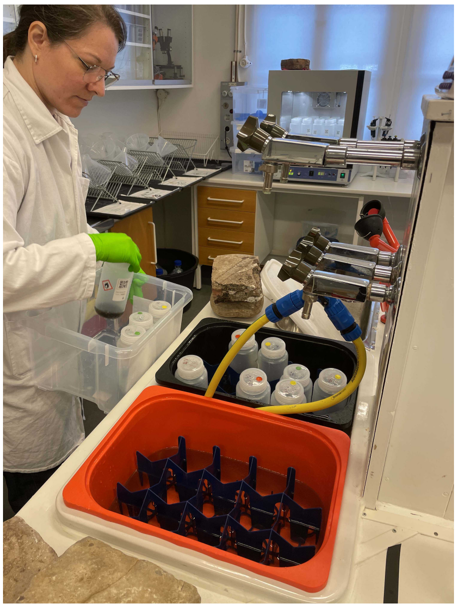

Water comes out of the tap at 60°C. Each water bath container (red and black in the photo) have a hole on the side from where water can flow out. This system keeps the water flowing inside the water bath container at a constant temperature of 60°C.

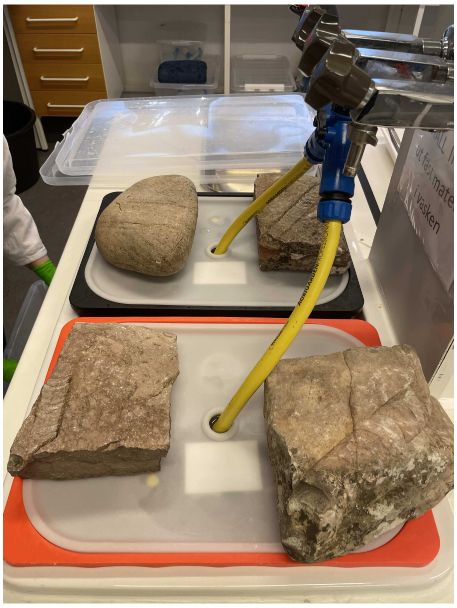

Water flows continuously from the tap into the two water bath containers. Double-stone Lock System (patent pending) - Secure and Hi-Tech ;)

12 **Incubate samples at 56°C in a shaking dry incubator**

12.1 Place the pre-heated insect bottles inside the shaking incubator.

12.2 Reduce the incubator temperature to 56°C and set it up to 90 rpm.

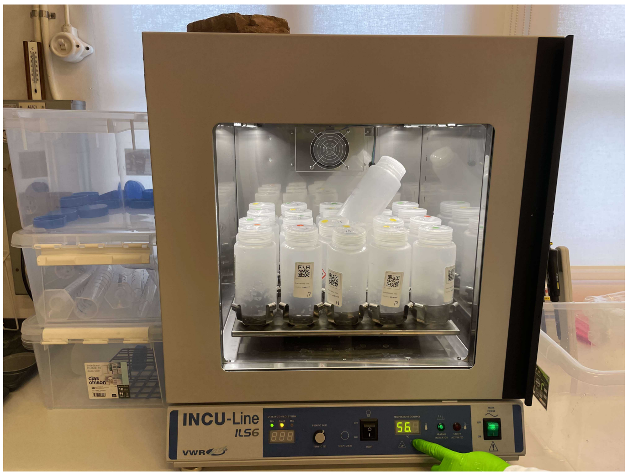

12.3 Incubate for 2 hours and 45 minutes.

13 **Measure EtOH concentration (OPTIONAL)**

Whilst samples are incubating, you can read the concentration of the preservative ethanol if you want to record this data.

13.1 At each station, open the ethanol receiving bottle and pour ca 5 mL of EtOH into a 10 mL beaker.

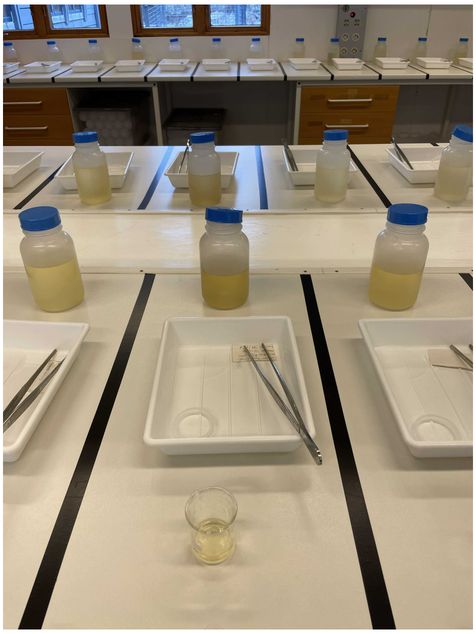

13.2 Measure the EtOH concentration using the density meter instrument and register values in the data sheet.

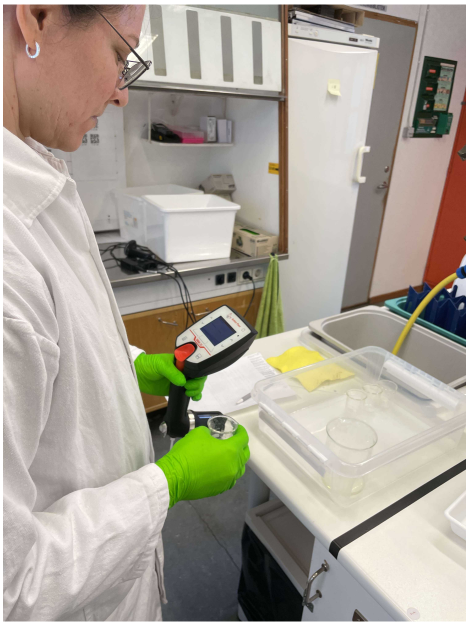

13.3 If EtOH concentration is lower than 90%, add 95% ethanol to the ethanol receiving bottle and repeat steps 13.1 and 13.2 until you get EtOH concentration >90%.

13.4 At the end, use a squirt bottle with bleach 10% solution and a paper towel to wipe all working station surfaces.

14 Decant lysate solution and preserve insect sample for long term storage

In the next steps we will decant the lysate solution to the lysate collecting bottle which will be stored at -20°C for long term storage.

14.1 Place one lysate collecting bottle (250 mL NALGENE HDPE wide mouth) and one lysate microtube (2 mL microtube with screw cap) at each working station and label them accordingly. Lysate bottles should be 500 mL capacity only if samples are greater than 200 mL in volume after adding the lysis buffer.

14.2 Remove insect bottles from the dry incubator and move each insect bottle back to its respective working station.

14.3 Starting with working station 1, open the insect bottle and place the second nylon mesh circle over the bottle opening.

14.4 Secure the mesh in place using the bottle seal with the help of the large forceps.

14.5 Pour the lysate from the insect bottle slowly into the labelled lysate collecting bottle.

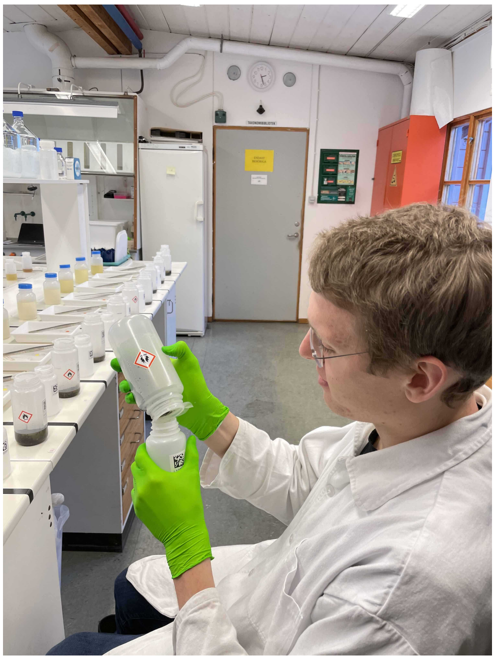

Decanting lysate from insect bottle into qr-coded lysate collecting bottle.

14.6 Wait until dripping has stopped and close the lysate collecting bottle.

14.7 For the insect bottle, remove the bottle seal and push the nylon mesh into the bottle with the help of the large forceps.

14.8 Add the archive paper label that was removed from the insect bottle in step 7.1 (if you have one) and pour the ethanol that was decanted in step 7.4 into the insect bottle and close it.

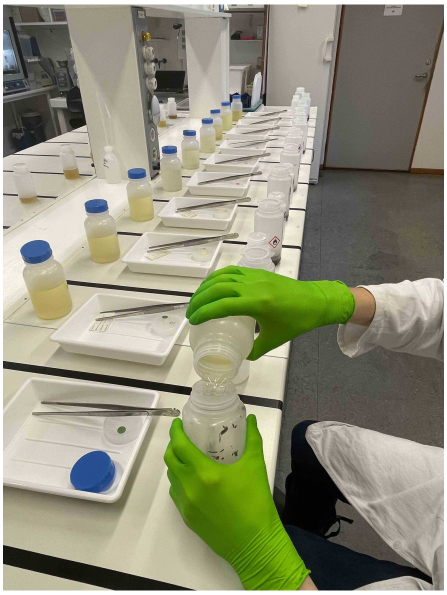

Adding the original preservative EtOH back into the insect bottle for long term storage.

14.9 Repeat steps 14.3-14.8 for every sample.

14.10 Place the trays, ethanol retrieving bottles, bottle seals and forceps in the 10% bleach container at the washing station. Make sure to move all items to the soap container after a minimum of 15 minutes.

15 **Take aliquot of the lysate for DNA purification (optional)**

In the next steps we will transfer a 1.8 mL aliquot of the lysate to the 2 mL lysate microtube which will be used as a working stock for DNA purification. If you will proceed with DNA purification straight away you can save time by transferring the precise aliquot of lysate (225 µL if you use step 16 below or 390 µL if you use the alternative option 17) directly from the lysate bottle into a deep well plate and proceed with DNA purification from step 16.2 or step 17 onwards.

15.1 Gather a 1000 µL micropipette, one box of 1000 µL filtered tips and a 96 well microtube rack.

15.2 Gently shake the lysate bottle to homogenize the liquid inside before taking the aliquots.

15.3 Cycle through each station and transfer 2x900 µL aliquots of lysate from each lysate bottle into its corresponding lysate microtube.

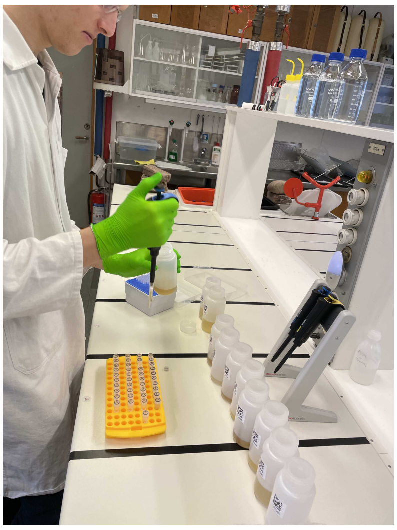

15.4 Fill in the microtube rack with the lysate microtubes starting with A1 to A12, B1 to B12, and so on. Leave the last four wells (H9; H10; H11 and H12) empty as these will serve as negative (H9) and positive (H10) DNA purification controls and negative (H11) and positive (H12) library preparation controls. Label each 96-well microtube rack with a unique label.

15.5 The lysate bottles and lysate aliquots in the microtube racks should be stored at -20°C for long term preservation if not used straightaway.

SECTION 3: DNA Purification

16 **DNA purification using King Fisher Flex 96 robot**

For our project we purify a 225 µL aliquot of lysate from each sample using silica-coated magnetic beads with the KingFisher Cell and Tissue DNA kit on a King Fisher Flex 96 robot (both Thermo Scientific) according to manufacturer instructions. You can use alternative kits in your project but make sure you optimize the amount of lysate input needed to obtain enough DNA for your purposes. If you don't have a King Fisher Flex 96 robot, you can purify your DNA manually (see our proposed alternative DNA purification method in step 17 below) but this will take substantially longer to process.

16.1 Remove the 96-well microtube rack from the freezer and let it thaw at room temperature. To speed up this process the microtube rack can be added to a shaking incubator at 37°C. Make sure lysates are completely thawed and well mixed before proceeding into the next steps.

16.2 Open each lysate microtube and transfer 225 µL lysate aliquot from each microtube into a 96 deep-well plate (2.2 mL capacity). This step can be speed up considerably if you use an 8-multichannel adjustable pipette that allows transferring multiple samples from racks to 96-well plates.

16.3 In well H9 add 225 µL of ddH2O and in well H10 add 225 µL of a lysate sample with known DNA concentration to act as a positive control. Leave well H11 and H12 empty to serve as

positive and negative controls for the library preparation steps.

16.4 Follow your kit specific instructions. We recomend eluting the purified DNA with 100 µL of elution buffer or TE buffer. (Note that buffers containing EDTA, such as TE, can inihbit some polymerases. Qiagen Multiplex polymerase that we recommend in Library Preparation steps works well with TE but if you plan to experiment with different polymerases, then you're safer using purified water to elute DNA).

16.5 Transfer 40 µL of purified DNA to a 96-well PCR plate for long term storage and the remaining 40 µL to another 96-well PCR plate that will be used as working DNA stock for library preparation in step 18 below.

16.6 Once DNA is purified measure DNA concentration using Qubit fluorometer instrument for the entire plate or for selected samples. In our project, for each plate, we measure concentration for samples A8, B8, C8, D8, E8, F8, G8, H8 plus the negative (H9) and positive control (H10) only.

17 **DNA purification using homemade magnetic beads - (ALTERNATIVE OPTION)**
Before proceeding with this step, prepare Magnetic Bead Solution as described in "Materials" section.

17.1 Prepare fresh aliquots of 80% EtOH.

17.2 Add 585 µL (see Note 8) of Magnetic Bead Solution to each well of a 96 deep-well plate (2.2 mL capacity). To speed up this process, you can add the Magnetic Bead Solution first to a 100 mL reagent reservoir and use an 8-or-12 multichannel pipette to dispense the beads into the 96 deep-well plate. Leave well H11 and H12 empty as these will be use as negative and positive controls during library preparation in step 18 below.

17.3 To each well add 390 µL of lysate from each sample and mix by pipetting up and down. In well H9 add the 390 µL of ddH20 (negative control) and in well H10 add 390 µL of a lysate sample with known DNA concentration to act as a positive control..

Note 8: The amount of input lysate and Magnetic Bead Solution will depend on your samples and the desired DNA concentration. Usually this has to be optimized beforehand. We use a ratio of Magnetic Bead Solution to lysate between 1.5-2.0x. Be aware that the maximum amount of lysate and Magnetic Bead Solution that can be purified in a 96 deep-well plate cannot exceed 1000 µL, otherwise beads will not be drawn to the magnet.

17.4 Incubate mixture 5 min at room temperature.

17.5 Place the plate on plate magnetic stand for 5 min.

17.6 Whilst on magnetic stand, remove supernatant (use multichannel pipette).

17.7 Pour 80% EtOH into an 100 mL reagent reservoir and using a multichannel pipette add 975 µL of 80 % EtOH to each well.

17.8 Incubate on magnetic stand for 1 min.

17.9 Remove supernatant.

17.10 Repeat steps 17.7 – 17.9

17.11 Wait 5 min until dry (on magnetic stand).

17.12 Remove plate from magnetic stand, add 100 µL of TE x 1 using a multichannel pipette and mix by pipetting up and down. (You can use purified water instead of TE if you plan on using different polymerase).

17.13 Place the plate on magnetic stand for 2 min.

17.14 Transfer 40 µL of supernatant to a new 96-well plate for long term storage and the remaining 40 µL to another 96-well plate that will be used as working DNA stock for library preparation below.

SECTION 4: Library preparation and Sequencing

18 **Library preparation using a two step PCR approach**
For amplicon library preparation we use a two-step PCR approach. In the first step (PCR 1) we amplify the target gene: 418 base pairs of the mitochondrial cytochrome oxidase 1 (CO1). In the second step (PCR 2) we add the indexes to the amplified sequences and fill up Illumina adapters. Before proceeding with this step prepare primer mix solutions for forward and reverse primers as described in "Materials" section.

18.1 Set up PCR 1 by creating a master mix as described below. We carry out PCR 1 in a total volume of 40 µL. To prepare the master mix for a 96-well plate, multiply the volumes below by 95 (as one well of the plate is left empty for PCR 2 negative control). Be aware that in those 95 samples one should contain ddH2O instead of DNA (PCR 1 negative control).

A	B
Component	Volume (µL)
2x Qiagen Multiplex	20
10 µM CO1_BF3_P5 primer mix	4
10 µM CO1_BR2_P5 primer mix	4
ddH2O	8
+ DNA template	4

18.2 Run PCR 1 using the following conditions:
Note 9: All steps above should be performed in a pre-PCR room and steps below should be performed in a pos-PCR room. Make sure you have dedicated lab equipment for each room, such as pipettes, tips, racks, plate shakers, magnetic stands for plates and for microtubes, etc.

A	B	C	D
Step	Temp. [oC]	Time [s]	Cycles
Initial denaturation	95	900	1
Denaturation	94	30	25
Annealing	50	90	25
Extension	72	90	25
Final extension	72	600	1
Store	8	∞	

18.3 Run PCR 1 products on a 2,5% agarose gel (2 µL PCR product + 2 µL loading dye).

18.4 Clean up PCR 1 products by following the steps below. Make sure you prepare Magnetic Bead Solution as explained in the "Materials" section before starting this step.

1. Prepare fresh aliquots of 80% EtOH.
2. Transfer 20 µL of PCR1 product to a 96 deep-well plate and add 40 µL (x 2.0) of Magnetic Bead Solution, and mix by pipetting (or vortex the entire plate using a plate shaker).
3. Incubate mixture for 5 min at room temperature.
4. Place the plate on a magnetic stand for 2 min.
5. Remove supernatant (use multichannel pipette).
6. Pour alcohol into a 100 mL reagent reservoir and, using a multichannel pipette, transfer 150 µL of 80% EtOH to each well.
7. Incubate on a magnetic stand for 1 min.
8. Remove supernatant.
9. Repeat steps 6 – 8 above.
10. Wait 5 min until the magnetic bead pellet gets dry (on magnetic stand).
11. Remove samples from the magnetic stand, add 20.5 µL of TE buffer and mix by pipetting up and down. It is good to centrifuge the plate before next step.
12. Place back the plate on the magnetic stand for 2 min.
13. Transfer supernatant (20 µL) to a new 96-well plate.
14. Mark the plate with a unique identifier and store at 4°C (for a few days) or at -20°C (for a longer period).

18.5 Prepare 10 µM solution of the index i5 primer and index i7 using TE buffer as dilution solution.

18.6 Set up PCR 2 (indexing) in a total volume of 10 µL as described below, using 1 µL of PCR 1 product as input. To prepare the master mix for a 96-well plate, multiply the volumes below by 100. Be aware that in 96 samples one sample should contain ddH2O instead of DNA (PCR 2 negative control).

A	B

A	B
Component	Volume (µL)
2x Qiagen Multiplex	5
10 µM Index i5	1
10 µM Index i7	1
ddH2O	2
+ clean PCR 1 product	1

18.7 Run PCR 2 using the following conditions (* number of cycles depends on the gel band intensity):

A	B	C	D
Step	Temp. [oC]	Time [s]	Cycles
Initial denaturation	95	900	1
Denaturation	94	30	6 – 7*
Annealing	50	90	6 - 7*
Extension	72	90	6 - 7*
Final extension	72	600	1
Store	8	∞	

18.8 Run PCR 2 products on a 2,5% agarose gel (2 µL PCR 2 product + 2 µL loading dye).

19 **Pooling strategy and sequencing**

Because our inserts are ca. 462bp long (418bp region of interest, plus primers, plus variable-length inserts), we require a sequencing platform providing reads with total length >500 bp. NovaSeq SPrime 2x250bp flow cell is the most cost-effective solution at the time of publishing this protocol. We sequence 768 libraries (8x96 well plates) per NovaSeq SPrime 2x250bp flow cell: 384 samples (4x96-well plates) pooled per lane. Before submitting libraries for sequencing we create two Master Pools (A and B) composed of 384 libraries each. Each Master pool is be sequenced in 1 lane of a NovaSeq SPrime 2x250bp flow cell. According to Illumina specifications, the NovaSeq SPrime 2x250bp should provide up to 800M read pairs per flow cell, but in our experience, 900M or more reads were often obtained. This results in an average of >1M read pairs for each of 768 libraries sequenced per flow cell.

19.1 Create a pool of samples (libraries) from each 96-well plate by mixing PCR 2 products in an 1.5 mL microtube, based on gel band intensity:

- strong band - 1 µL
- medium band - 2 µL

- weak band - 4 µL
- no visible band - 8 µL

19.2 Clean the "plate pool" created above with Promega ProNex® Size-Selective Purification System. Follow the manufacturer's instructions, using 1/1.5 (v/v) pool to magnetic beads ratio. As standard we use 80 µL of pool for cleaning.

19.3 Measure DNA concentration of the "plate pool" on Qubit.

19.4 Create two Master Pools (A and B) by mixing together the "plate pools" obtained in step 18.9, based on the DNA concentration of each plate pool measured in step 18.11. Each Master Pool should contain 4 plates (384 libraries).

19.5 Clean each Master Pool by repeating point 19.2, but use 1/**1.3** (v/v) pool to magnetic beads ratio. Suspend the final product in TE x 0.1 (or ddH2O). Volume in which to suspend depends on desired DNA concentration.

19.6 Measure the DNA concentration of the cleaned Master Pools A and B on a Qubit fluorometer instrument following the manufacturer instructions.

19.7 Check library quality of each Master Pool A and B (e.g. lack of primer-dimmers) by running them on Bioanalyzer or Tapestation before submitting for sequencing.
In an unlikely event of detecting primer-dimers, repeat cleaning step as in point 19.5.
