# Supplementary material for: FAVIS: Fast and versatile protocol for non-destructive metabarcoding of bulk insect samples
Source: PLoS One. 2023 Jul 19;18(7):e0286272. doi: 10.1371/journal.pone.0286272 (PMC10356154; doi:10.1371/journal.pone.0286272)
Supplement: S2 File — (PDF) [file pone.0286272.s005.pdf]

Supporting Information for the "FAVIS: Fast And Versatile protocol for non-destructive metabarcoding of bulk Insect Samples"

Authors: Elzbieta Iwaszkiewicz-Eggebrecht, Piotr Łukasik, Mateusz Buczek, Junchen Deng, Emily A. Hartop, Harald Havnås, Monika Prus-Frankowska, Carina R. Ugarph, Paulina Viteri[1], Anders F. Andersson, Tomas Roslin, Ayco J. M. Tack, Fredrik Ronquist, Andreia Miraldo

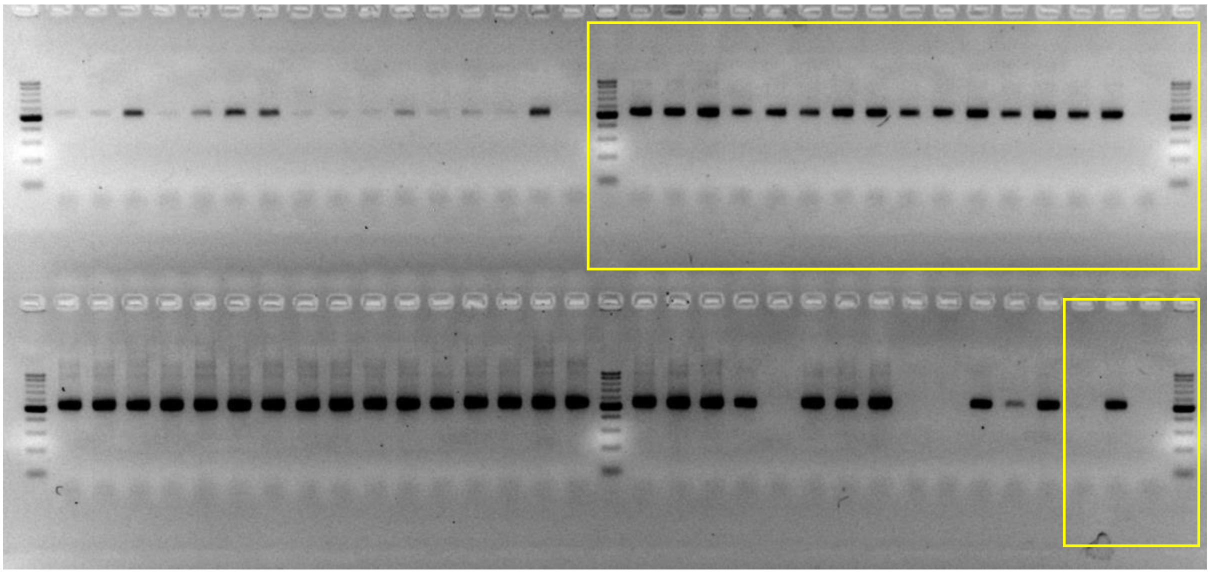

Figure S1. Original, uncropped electrophoresis gel picture underlying Fig. 2a from the main text. It shows products of the PCR I - length of the product was ca. 490bp. Yellow frame indicates which parts of the gel were presented in the main text. The Gel was 2.5% agarose and the DNA ladder used was Perfect Ladder 100-1000bp (EurX, Poland).

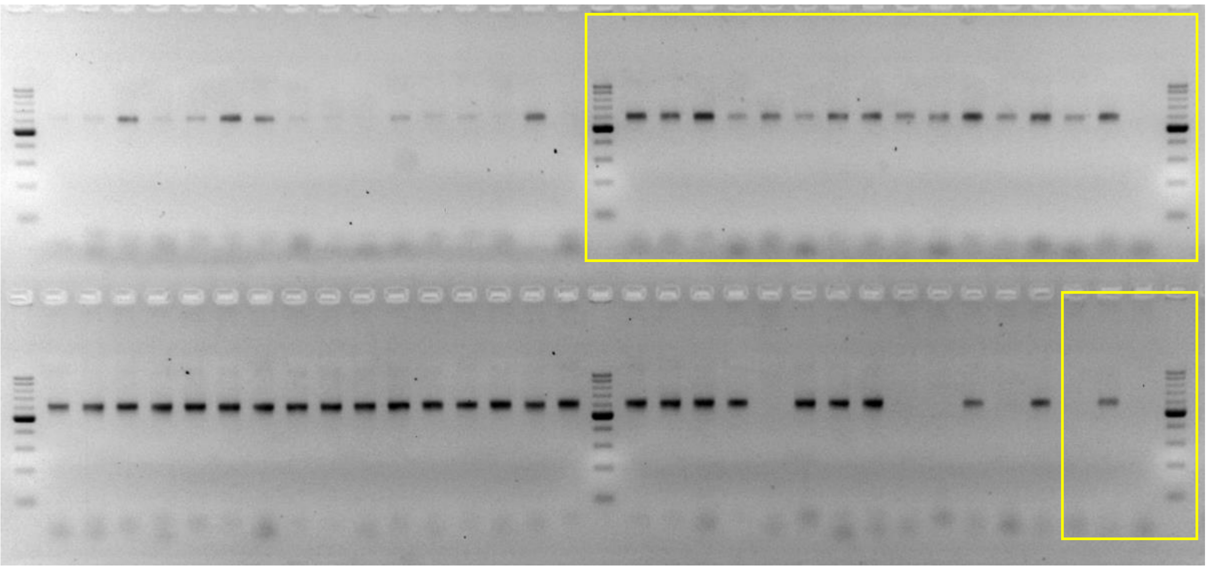

Figure S2. Original, uncropped electrophoresis gel picture underlying Fig. 2b from the main text. It shows products of the PCR II - length of the product was ca. 563bp. Yellow frame indicates which parts of the gel were presented in the main text. The Gel was 2.5% agarose and the DNA ladder used was Perfect Ladder 100-1000bp (EurX, Poland).

Table S1: Timeline for the processing of 184 samples using FAVIS protocol

Day	Sections	Steps		Duration (h)	Number of samples processed
day 1	section 1-2	step 1-15	Preparation and Sample processing (mild lysis)	8	40 samples + 1 negative control
day 2	section 1-2	step 1-15	Preparation and Sample processing (mild lysis)	8	40 samples + 1 negative control
day 3	section 1-2	step 1-15	Preparation and Sample processing (mild lysis)	8	40 samples + 1 negative control
day 4	section 1-2	step 1-15	Preparation and Sample processing (mild lysis)	8	40 samples + 1 negative control
day 5	section 1-2	step 1-15	Preparation and Sample processing (mild lysis)	6	19 samples + 1 negative control
day 5	section 3	step 16	DNA purification (using robot)	2	2x92 samples
day 6	section 4	step 18	Library preparation (PCR1 + PCR2)	8	2x92 samples
day 7	section 4	step 19	pooling*	8	2x92 samples

*If you use the same sequencing strategy as in FAVIS protocol you should only do this step when you have libraries corresponding to 8 plates. You can then create two Master pools, each composed of 4 plates.
